# Fatty acid metabolism-derived prognostic model for lung adenocarcinoma: unraveling the link to survival and immune response

**DOI:** 10.3389/fimmu.2025.1507845

**Published:** 2025-03-13

**Authors:** Rui-Ze Wu, Qian-Qian Sun, Yao Fu, Han-Nong Yu, Wei-Yang Liu, Yong-Hui Wu, Han Zhang, Yu-Lin Pan, Xin Rui

**Affiliations:** ^1^ School of Public Health, Harbin Medical University, Harbin, China; ^2^ Department of Medical Oncology, Harbin Medical University Cancer Hospital, Harbin, China; ^3^ School of Medicine and Health, Harbin Institute of Technology, Harbin, China

**Keywords:** lung adenocarcinoma, fatty acid metabolism, prognostic model, immune response, drug sensitivity analysis

## Abstract

**Background:**

Lung adenocarcinoma (LUAD) is one of the most common malignant tumors globally, characterized by poor prognosis and high mortality. Abnormal fatty acid metabolism plays a crucial role in LUAD progression. This study aims to develop a prognostic model based on fatty acid metabolism to improve the overall prognosis of LUAD.

**Materials and methods:**

Bioinformatics analyses were performed using TCGA and GEO datasets, supplemented by cell experiments. A total of 309 fatty acid metabolism-related genes were identified from MsigDB. Differentially expressed genes were analyzed using the ‘limma’ R package. A prognostic model was constructed using LASSO regression and validated with survival analyses via the ‘survminer’, ‘survival’, and ‘pROC’ R packages. The analysis included somatic mutations, tumor mutation burden, clinical correlations, stemness analysis, cytokine correlations, and enrichment analysis. Protein interaction networks were constructed using STRING and Cytoscape, while immune cell infiltration and immunotherapy responses were evaluated with the ‘oncoPredict’ R package. Results were validated through cell experiments and immunohistochemistry staining of lung tissues.

**Results:**

We identified 125 differentially expressed genes related to fatty acid metabolism, with 33 genes significantly associated with prognosis. Patients in the high-risk group had poorer overall survival and progression-free survival, and the risk score correlated with gender, N stage, clinical stage, and T stage. The risk score was also associated with cancer stem cells, with a significantly higher mRNAsi index in the high-risk group. Additionally, the risk score correlated with various cytokine expressions and showed significant enrichment in cell cycle pathways. Key genes like CDK1 were highly expressed in LUAD cell lines and validated in clinical samples. The low-risk group showed better responses to immune checkpoint inhibitors, with the risk score correlating with immune checkpoint gene expression.

**Conclusion:**

This study successfully established a novel prognostic model based on fatty acid metabolism, which provides valuable insights for the treatment of LUAD.

## 1 Introduction

Based on global cancer statistics for 2022, lung cancer (LC) remains the most prevalent malignant tumor, ranking first in incidence rate in the same year. Approximately 2.5 million new LC cases are reported globally annually, accounting for 12.4% of total tumor cases worldwide. Furthermore, LC is the leading cause to cancer-related fatalities, with an approximated 1.8 million deaths each year, representing 18.7% of all cancer-induced mortalities (1). Non-small cell LC accounts for approximately 85% of all LCs, with lung adenocarcinoma (LUAD) emerging as the most prevalent subtype (2). LUAD is frequently characterized by nonspecific early symptoms, which leading to delayed diagnosis in some patients until advanced stages. Additionally, those with negative driver gene mutations have a markedly higher likelihood of experiencing recurrence and metastasis (3). The primary therapeutic approaches for LUAD consist of surgery, radiotherapy, chemotherapy, and targeted therapy (4, 5). These treatments are often combined in multimodal strategies, showing significant advancements in recent years (6). Nanomaterials have shown great potential in the early screening, diagnosis, treatment, and post-treatment monitoring of minimal residual disease (MRD) in lung cancer, providing new opportunities to enhance therapeutic outcomes. However, despite ongoing advancements in these methods and technologies, treatment efficacy remains suboptimal (7). Due to the molecular and pathological heterogeneity displayed by cancer cells throughout tumor progression, gene mutations, expression levels, and recombination events may vary among patients, leading to differences in tumor development, metastatic potential, and drug resistance. This variability greatly complicates treatment efforts, highlighting the need for effective tumor-targeting drugs (8).

Metabolic reprogramming refers to the process by which tumor cells alter metabolic pathways to accommodate their rapid proliferation and survival requirements. It is vital in the pathogenesis and progression of LUAD, furnishing the energy and materials essential for tumor cell proliferation and survival. The amplification of the glycolysis pathway, commonly known as the Warburg effect, has attracted significant attention in this context. This hypothesis, introduced by Otto Heinrich Warburg, posits that LUAD cells typically exhibit enhanced glycolysis pathway, favoring energy (adenosine triphosphate, ATP) production via glycolysis, even when oxygen is available. Such a metabolic arrangement enables cancer cells to swiftly produce ATP and create metabolic intermediates necessary for cell growth and division (9). A strong connection exists between glycolysis and fatty acid metabolism (FAM), as the metabolic intermediates and energy generated through glycolysis are essential for fatty acid synthesis. Simultaneously, the end products and energy status from FAM provide feedback to regulate the glycolysis pathway (10). As a result, alterations in FAM significantly impact on the initiation and advancement of LUAD (11). Growing evidence suggests that FAM undergoes modifications within LUAD tissues (12), influencing the types, quantities, and regulatory mechanisms of lipid signaling molecules (13). FAM undergoes regulation not solely through internal oncogenic pathways but also via the tumor microenvironment (TME), which comprises various cell types, cytokines, growth factors, DNA, RNA, and nutrients, including lipids (14). Additionally, aberrant FAM can disrupt oncogenic signaling pathways in cancer cells and influence nearby normal cells by secreting components, including lipids (15). Nonetheless, the characteristics and functions of FAM-related genes in LUAD remain insufficiently investigated.

**Figure 1:**
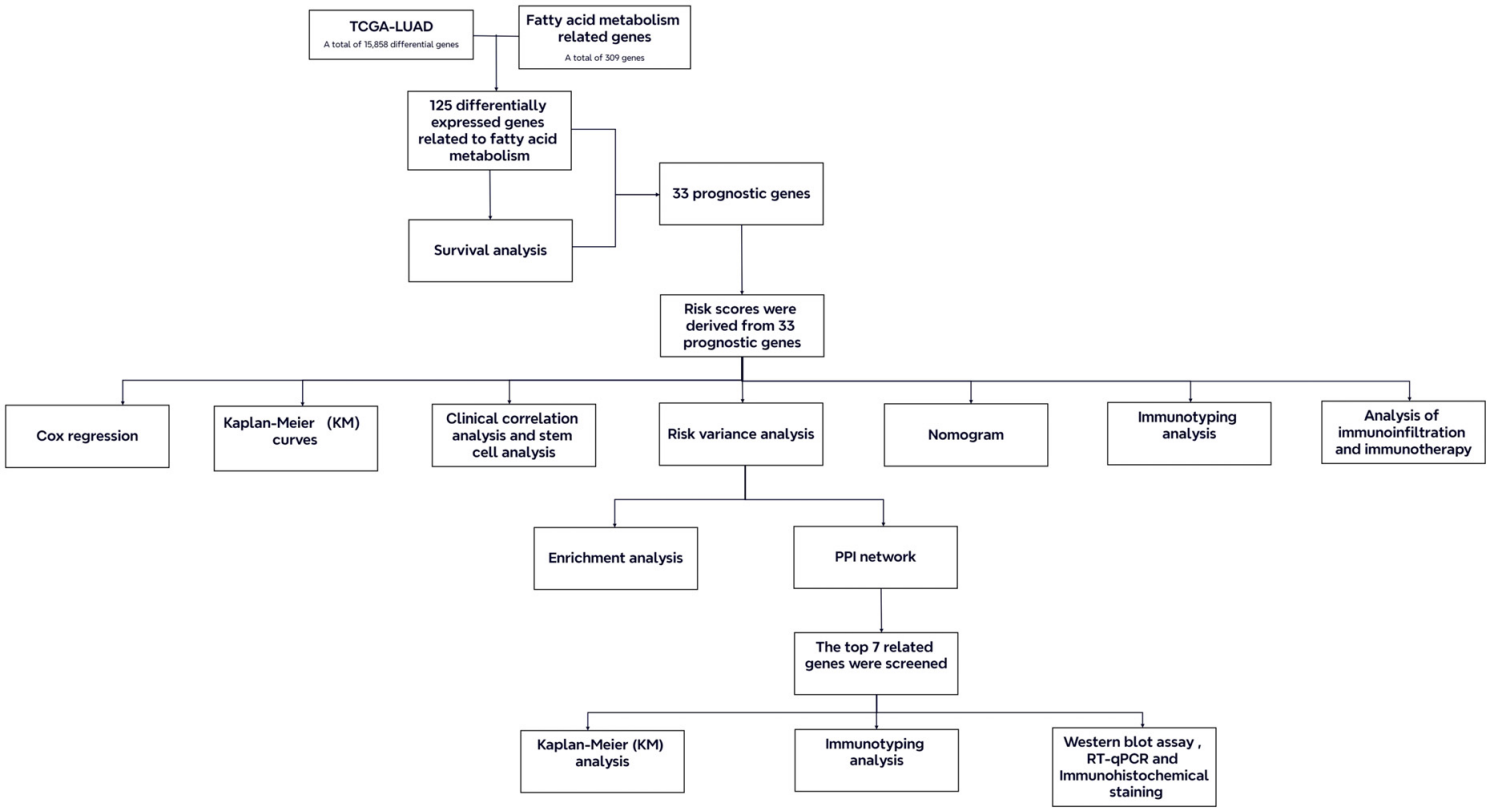
Flow chart of the study.

Current research suggests that the overexpression of fatty acid binding protein 5 has been correlated with a poor prognosis in LUAD, indicating its potential as a novel target for therapeutic target (16). The downregulation of fatty acid synthase has been demonstrated to impede the progression of LUAD by modulating glucose metabolism and suppressing the AKT/ERK signaling pathway (17). Liang et al. suggested that overexpression of the FAM enzyme-related genes influences the growth, differentiation, and metastasis of LUAD cells through various signaling pathways (18). These results illustrate that dysregulated FAM significantly impacts on the development of LUAD. Hence, identifying and validating FAM-related genes that exhibit significant differences between LUAD patients and healthy individuals is essential for formulating novel prognostic models and refining clinical therapeutic approaches. In this study, we focused on the alterations of FAM in LUAD patients. We utilized publicly available clinical and whole-genome data to construct and validate a LUAD prognostic model based on FAM-related genes. Comprehensive analyses and experimental validations were carried out on the model and key genes to promote the development of clinical treatments for LUAD (**Figure 1**).

## 2 Methodologies and materials

### 2.1 Dataset and preprocessing

RNA sequencing data and clinical details of LUAD patients were retrieved from The Cancer Genome Atlas (TCGA) database (https://www.cancer.gov/tcga/). Following the exclusion of patients lacking survival data, a total of 524 LUAD samples, along with 58 adjacent normal tissue specimens, were utilized. For validation purposes, the GSE68465 dataset was gathered from the Gene Expression Omnibus (GEO) database (https://www.ncbi.nlm.nih.gov/geo/). This dataset provides extensive gene expression profiles and survival data specific to LUAD patients. The initial gene expression information was available in CEL files, which also contained corresponding probe data.

### 2.2 Identification of FAM-related genes and differentially expressed genes

FAM-related genes were sourced from the Molecular Signature Database (https://www.gsea-msigdb.org/gsea/msigdb/), resulting in the identification of 309 relevant genes. Differential gene expression analysis between LUAD and adjacent normal tissues was executed within the TCGA dataset employing the ‘limma’ R package, applying |log2FC| > 0.585 and *P* < 0.05 as the filtering thresholds. The intersection between the FAM-related genes and the DEGs in LUAD was then determined, enabling the identification of FAM-related DEGs for further investigation.

### 2.3 Development of prognostic risk model

Univariate Cox regression analysis was applied to the screened FAM DEGs utilizing the ‘survival’ R package to ascertain genes linked to prognosis. Subsequently, the LASSO regression technique was employed via the ‘glmnet’ R package to reduce overfitting and develop the prognostic model. Risk scores (RS) for individual patients were computed based on the regression coefficients (β) obtained from LASSO regression in combination with gene expression levels. The computation formula is presented below:


$$\text{Risk Score}=\sum_{i=1}^{n}\text{Coefficient }{\left(\text{β}\right)}_{\text{i}}{*\text{X}}_{\text{i}}$$

Subjects were categorized into high-risk and low-risk cohorts utilizing the median RS. Survival analysis was then executed employing the ‘survminer’ and ‘survival’ R packages, while receiver operating characteristic (ROC) curves were produced via the ‘pROC’ R package to evaluate the model’s prognostic value. The stability of the model was subsequently confirmed using the GEO dataset (GSE68465).

### 2.4 Mutation types analysis

The somatic mutation data for LUAD patients were obtained from the TCGA database (https://www.cancer.gov/tcga/). Disparities in somatic mutation profiles between high-risk and low-risk cohorts were assessed and visualized through a waterfall plot created utilizing the ‘maftools’ R package.

Tumor mutation burden (TMB), characterized as the number of tumor mutations per megabase in individual specimens, was computed by applying the ‘TMB’ function from the ‘maftool’ R package and subsequently underwent a logarithmic transformation to facilitate visualization.

### 2.5 Clinical relevance analysis

Clinical data were integrated with the RS of LUAD patients, and clinical correlation analyses were conducted for gender, age, N stage, T stage, M stage, and overall clinical stage using the ‘ggpubr’ R package. The outcomes were visualized through box plots. A nomogram was subsequently established, along with calibration curves, based on clinical characteristics, RS, and survival times.

### 2.6 Coherence analysis

The stemness index (mRNAsi score) was procured from stem cell gene expression data obtained from TCGA-LUAD patients utilizing the ‘TCGAbiolinks’ R package. A scatter plot was utilized to depict the link between each patient’s RS and stemness index. Following this, differential and correlation analyses were conducted, with the corresponding results visualized.

### 2.7 Correlation analysis of cytokines

The cytokine gene catalog was procured from the NCBI gene database (https://www.ncbi.nlm.nih.gov/gene/), and the corresponding expression data were procured from the TCGA-LUAD dataset. Subsequently, these data were classified into high-risk and low-risk cohorts on the basis of RS, facilitating differential expression analysis, followed by the generation of a heatmap. Additionally, a CA between the RS of LUAD patients and cytokine expression levels was conducted, leading to the creation of a correlation scatter plot with trend lines.

### 2.8 Enrichment analysis

The ‘clusterProfiler’ R package was employed to conduct Gene Ontology (GO), Kyoto Encyclopedia of Genes and Genomes (KEGG), and gene set variation analysis (GSVA) enrichment analyses on the identified risk genes. A significance criterion of *p*-value < 0.05 and *q*-value < 0.05 was employed to pinpoint pathways exhibiting significant enrichment. This approach was aimed at uncovering the principal enriched signaling pathways and biological functions between the high-risk and low-risk cohorts.

### 2.9 Protein-protein interaction network

PPIs among the extracted genes were predicted using the STRING database, with the resulting network visualized through Cytoscape 3.10.1 (https://cytoscape.org). The Cytohubba plugin in Cytoscape was subsequently utilized to identify the top 7 genes with the strongest correlations.

### 2.10 Immune cell infiltration analysis and immunotherapy analysis

The CIBERSORT algorithm (with 1,000 permutations) was employed to evaluate the prevalence of 22 tumor-infiltrating immune cell types within the gene expression matrix of the TCGA-BRCA dataset, and the findings were visualized via a heatmap. Furthermore, the Wilcoxon test was utilized to assess the expression levels of immune checkpoints between high- and low-risk cohorts, aiming to anticipate the potential impact of immunotherapy on the basis of survival analysis outcomes. Immunotherapy analysis was carried out using The Cancer Immunome Atlas (TCIA) (https://tcia.at). For drug sensitivity analysis, the ‘oncoPredict’ R package was employed to estimate the half-maximal inhibitory concentration (IC50) values for each chemotherapeutic agent.

### 2.11 Patients and tissue samples

All patients were hospitalized at the Third Affiliated Hospital of Harbin Medical University and diagnosed through pathological examination. Pathological diagnoses were made per the 8th edition of the American Joint Committee on Cancer guidelines. Informed consent was procured from all participants, and the study protocol was sanctioned by the Internal Audit and Ethics Committee of the Third Affiliated Hospital of Harbin Medical University.

### 2.12 Cell lines and cell culture

The Beas-2B, A549, PC9, and H1299 CLs were procured from the Stem Cell Bank of the Chinese Academy of Sciences. Beas-2B and H1299 cells were cultivated in DMEM medium, supplemented with 80 U/L penicillin and 0.08 mg/mL streptomycin, while A549 and PC9 cells were kept in RPMI-1640 medium comprising identical antibiotic concentrations. All media were enriched with 10% fetal bovine serum. The CLs were kept under controlled conditions at 37°C, with 5% CO_2_ and 99% relative humidity.

### 2.13 Real-time quantitative polymerase chain reaction experiment

Total RNA from cells was procured utilizing a column-based RNA extraction kit (RC112-01, Nanjing Vazyme Biotech Co., Ltd., Nanjing, China). The isolated RNA was then converted to cDNA via reverse transcription (R202-02, Xinbei (Shanghai) Biotechnology Co., Ltd., Shanghai, China). Following PCR amplification (Q204-01, Xinbei (Shanghai) Biotechnology Co., Ltd., Shanghai, China), the RNA quantities were standardized in relation to β-actin RNA employing the relative Ct technique. The primer sequences are depicted in **Supplementary Table S1**.

### 2.14 Western blot analysis

Beas-2B, A549, PC9, and H1299 CLs underwent lysis in a chilled buffer containing 1% phenylmethylsulfonyl fluoride for 40 min. The proteins were subsequently denatured through heating in a 100°C water bath for 10 min and subsequently subjected to electrophoresis. Following this, primary antibody incubation with anti-cyclin-dependent kinase 1 (CDK1) (1:1000, Affinity Biosciences, Jiangsu, China) was carried out overnight at 4°C. Secondary antibody incubation followed for 1.5 h at 37°C (1:10000, Abclone, Wuhan, China). The antibody interactions were detected using an enhanced chemiluminescence kit (Wanlei, Shenyang, China).

### 2.15 Immunofluorescence staining

Tissue slides were exposed to primary antibodies against CDK1 (Wanleibio, Shenyang, China, WL02373, 1:1500), budding uninhibited by benzimidazoles 1 (BUB1) (Solarbio, Beijing, China, K111585P, 1:300), Cyclin A2 (CCNA2) (Solarbio, Beijing, China, K009488P, 1:300), Cyclin B1 (CCNB1) (Solarbio, Beijing, China, K000505P, 1:300), cell division cycle 20 (CDC20) (Solarbio, Beijing, China, K108719P, 1:300), discs large-associated protein 5 (DLGAP5) (Solarbio, Beijing, China, K111149P, 1:300), and TTK (Solarbio, Beijing, China, K007879P, 1:300) overnight at 4°C. Subsequently, secondary antibody treatment was applied, and DAB staining, along with hematoxylin counterstaining, was performed.

### 2.16 Statistical analysis

Statistical analyses were executed utilizing R4.3.1. Spearman and Pearson correlation analyses were applied to evaluate the links between RS, gene expression levels, immune scores, immune infiltration, and immune cell populations. Kaplan-Meier survival analysis was generated to contrast survival outcomes across diverse cohorts. The model’s predictive capability was assessed via ROC curve analysis. A *P*-value below 0.05 was deemed statistically significant.

## 3 Results

### 3.1 Identification of DEGs related to FAM in LUAD

**Figure 2:**
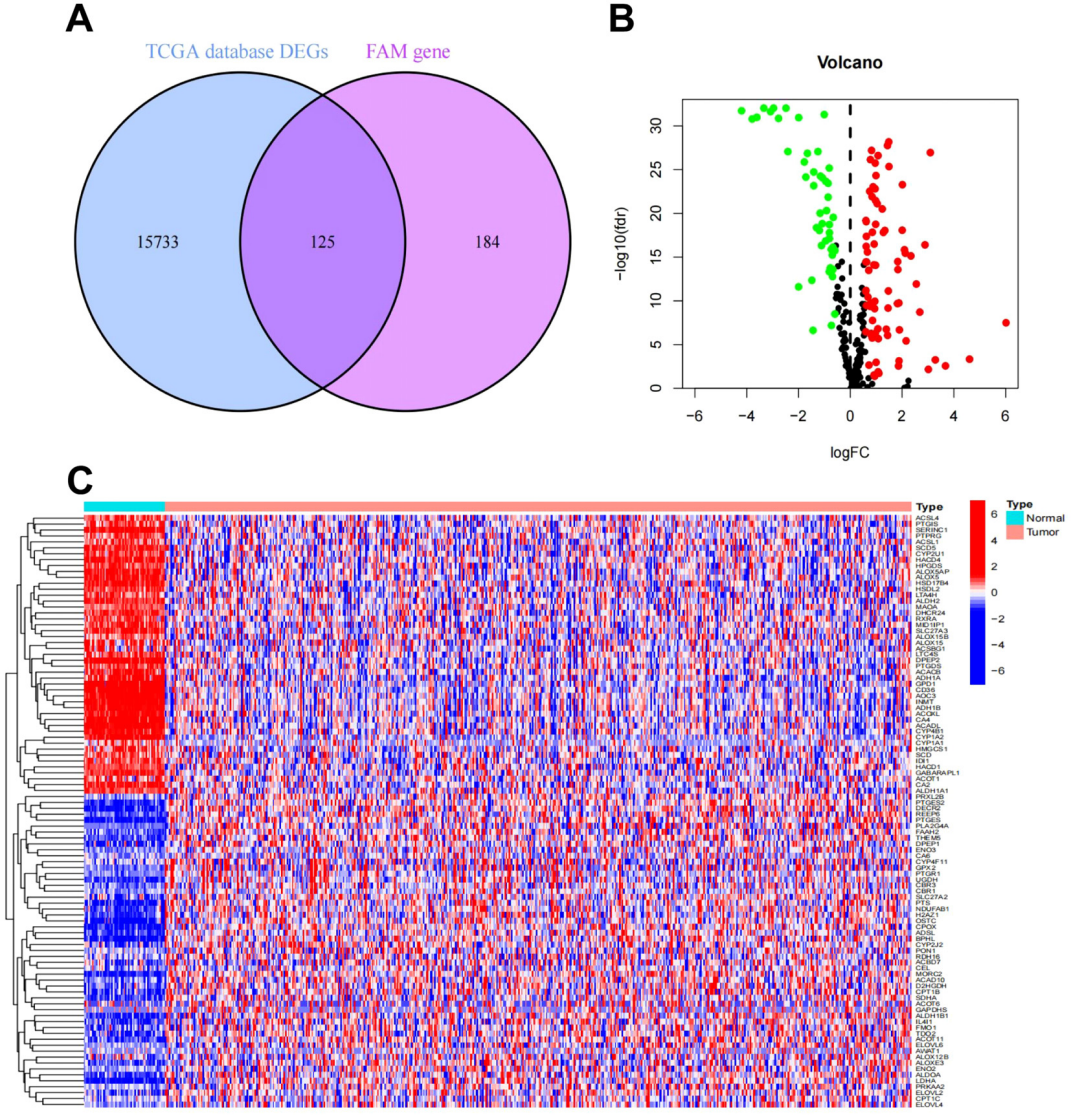
Differential expression analysis of FAM-related genes in LUAD. **(A)** Venn plot of FAM-related DEGs in the TCGA-LUAD cohort. **(B)** Volcano plot of FAM-related DEGs in the TCGA-LUAD cohort. **(C)** Heatmap of FAM-related DEGs in the TCGA-LUAD cohort.

To screen out the differentially expressed FAM-related genes in LUAD patients and provide key gene data for subsequent exploration of the association between LUAD and FAM as well as research on potential mechanisms. Genome-wide data, encompassing 524 LUAD samples along with 58 corresponding adjacent normal tissue specimens, were sourced from the TCGA-LUAD cohort. A differential expression analysis revealed 15,858 DEGs. These DEGs were subsequently cross-referenced with FAM genes. As depicted in **Figure 2A**, 125 overlapping genes were identified, comprising 78 genes that were upregulated and 47 that were downregulated. **Figures 2B, C** illustrate the heatmap and volcano plot, respectively, showcasing the differential expression of FAM-related genes. From these, the top 50 FAM genes with the greatest |log2FC| values were chosen to evaluate their correlations. The heatmap, presented in **Supplementary Figure S1A**, clearly underscores the division of the FAM genes into two distinct clusters.

### 3.2 Survival analysis of FAM-related DEGs in LUAD and construction of prognostic model

**Figure 3:**
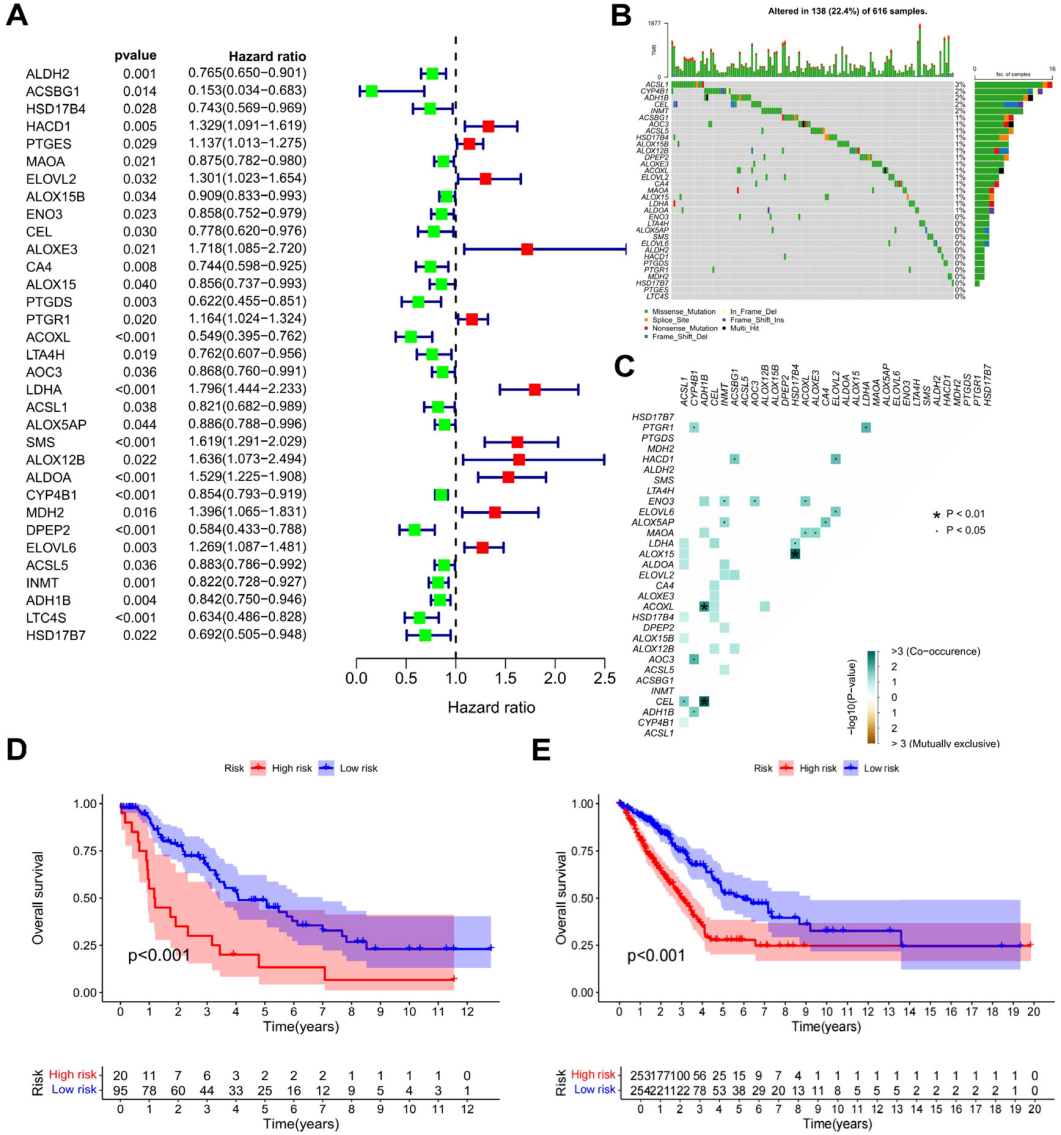
Survival analysis and prognostic model of FAM-related DEGs. **(A)** Forest plot of DEGs linked to patient prognosis (*P* < 0.05). **(B)** Waterfall plot of prognostic genes. **(C)** Co-mutation plot of prognostic genes. **(D)** OS curves for high-risk and low-risk patients in the TCGA dataset. **(E)** PFS curves for high-risk and low-risk patients in the TCGA dataset.

To provide a quantitative tool and basis for the evaluation of the prognosis of LUAD patients, we constructed a prognostic model. The gene expression patterns of FAM-related DEGs from the TCGA-LUAD cohort were combined with corresponding survival data. Univariate Cox regression analysis was executed on each gene, and those markedly associated with prognosis were identified and illustrated in a forest plot (*P* < 0.05). As depicted in **Figure 3A**, 33 genes related to prognosis were identified, including 11 classified as high-risk and 22 as low-risk. CA of prognostic gene mutations was subsequently performed (**Figures 3B, C**), revealing that the majority of alterations were missense mutations, with ACSL1 showing the highest mutation frequency. To develop a prognostic model, the TCGA-LUAD dataset was utilized as the “training set”, while the GEO dataset was employed as the “test set” (**Supplementary Table S2** and **Supplementary Figure S2**). RS and median risk values were calculated, enabling classification into high-risk and low-risk cohorts for survival analysis. As depicted in **Figures 3D, E**, and **Supplementary Figure S3A**, patients with elevated RS in both the TCGA and GEO datasets exhibited poorer overall survival (OS) and progression-free survival (PFS) (*P* < 0.001). Independent univariate and multivariate prognostic analyses further confirmed that the “RS” was an independent predictor of OS (**Supplementary Figures S3B, C**). A combined ROC curve, integrating RS with other clinical features, was plotted, with the RS displaying the highest AUC value of 0.683 (**Supplementary Figure S3D**). An additional ROC curve was generated to predict LUAD patients survival using the RS, as shown in **Supplementary Figure S3E**, with AUC values of 0.729, 0.713, and 0.683 for estimating 1-year, 3-year, and 5-year survival, respectively. These findings strongly indicate that the prediction model established in this investigation effectively predicts LUAD prognosis.

### 3.3 Clinical relevance and coherence analysis of the LUAD prognostic model

To explore possible differences in RS among patients with differing characteristics, survival data were examined for clinical correlations and represented using box plots, as illustrated in **Supplementary Figure S4**. Significant associations between RS and gender (*P* = 0.021), N stage (*P* < 0.001), clinical stage (*P* < 0.001), and T stage (*P* < 0.001) were identified. A nomogram was constructed by integrating RS with clinical variables to estimate 1-, 3-, and 5-year survival rates (**Supplementary Figure S5A**). The calibration plot revealed a robust alignment between the predicted survival rates from the nomogram and actual outcomes (**Supplementary Figure S5B**). Following this, an independent prognostic analysis of the nomogram was carried out. As depicted in **Supplementary Figures S5C, D**, the nomogram showed a *P*-value below 0.001, signifying that it could serve as an independent prognostic indicator for patient survival, separate from other clinical factors.

An expanding corpus of research indicates that cancer stem cells (CSCs), as a pivotal subset of cancer cells, markedly enhance tumor cell heterogeneity (19). mRNAsi data from LUAD patients were acquired to assess the mRNAsi index across different risk cohorts (**Supplementary Figure S6A**). The juxtaposition of mRNAsi indices between high- and low-risk cohorts, illustrated in **Supplementary Figure S6B**, revealed that the high-risk cohort had a substantially elevated mRNAsi index relative to the low-risk cohort. To further explore the association between the RS and the mRNAsi index, a correlation plot was created (**Supplementary Figure S6C**), which revealed a substantial positive association between these two factors. This finding further highlights the strong prognostic value of the constructed risk model.

### 3.4 CA between RS and cytokines

**Figure 4:**
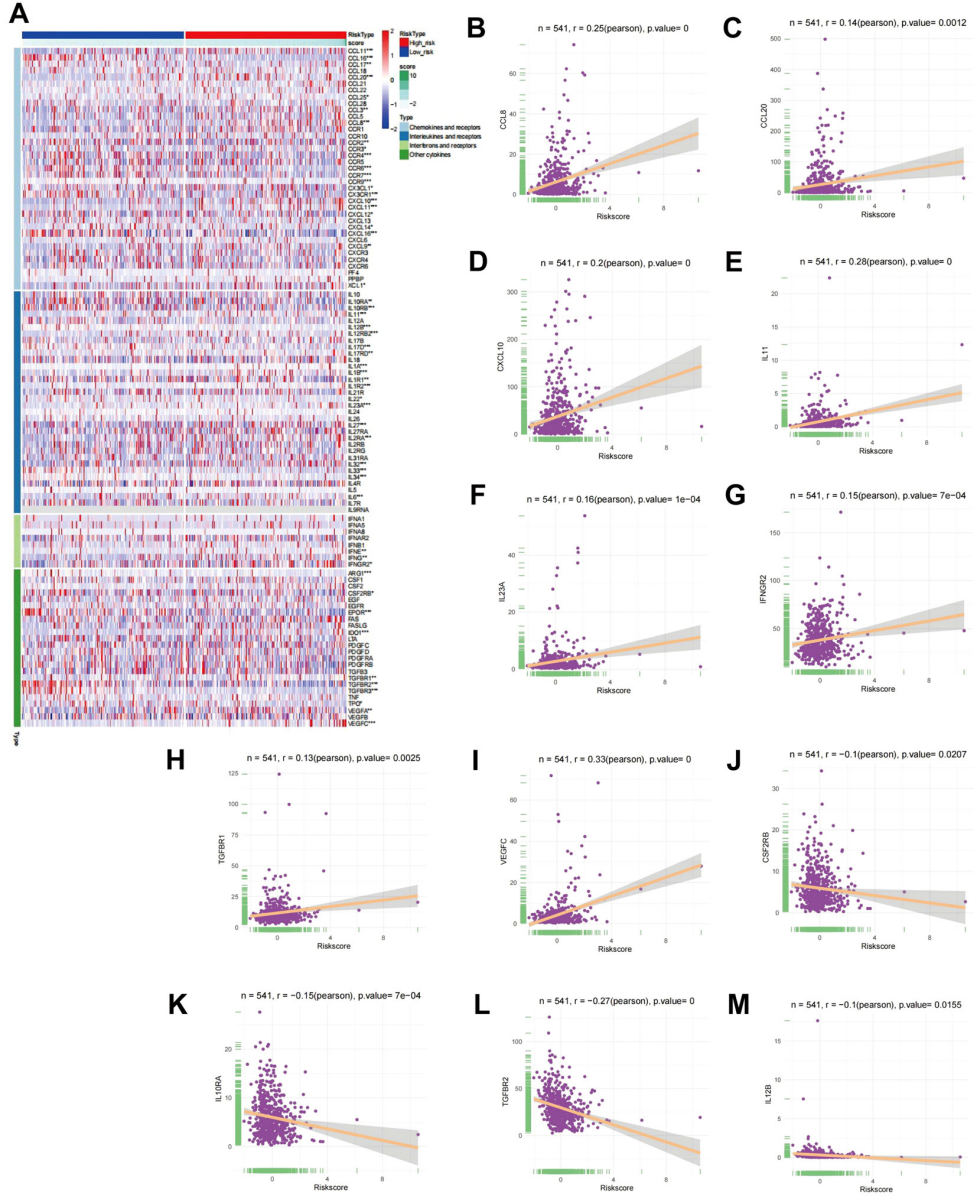
CA between RS and cytokines. **(A)** Heatmap showing differential expression of cytokines in high- and low-risk cohorts. **(B-M)** Scatter plots depicting the link between RS and the expression levels of CCL8, CCL20, CXCL10, IL-11, IL-23, IFNGR2, TGFBR1, VEGFBR1, CS3CR1, IL-10, TGFBR, and IL-12B.

Cytokines are known to have a crucial function in tumor initiation, advancement, spread, and immune modulation (20, 21). Investigating cytokines in detail can aid in the development of innovative therapeutic approaches for tumors. Consequently, an examination of the link between RS and cytokines was executed. Initially, cytokine and receptor expression levels were contrasted between high-risk and low-risk cohorts, and a heatmap illustrating the differential expression was produced (**Figure 4A**). The findings indicated notable variations in the expression of various chemokines (such as CCL11, CCL16, CCR2, CX3CL1), interleukins (such as IL11, IL12B, IL12RB2), interferons (IFNs) (such as IFNE, IFNG, IFNGR2), and other cytokines (such as ARG1, CSF2RB, EPOR, IDO1) and their corresponding receptors across the two cohorts. To further elucidate the relationship between cytokines and RS, a CA was performed, accompanied by correlation scatter plots. The analysis demonstrated that CCL8, CCL20, CXCL10, IL-11, IL-23, IFNGR2, TGFBR1, and VEGFBR1 were positively correlated with RS, while CS3CR1, IL-10, TGFBR, and IL-12B showed negative correlations with RS (**Figures 4B-M**).

### 3.5 Risk difference analysis and enrichment analysis

**Figure 5:**
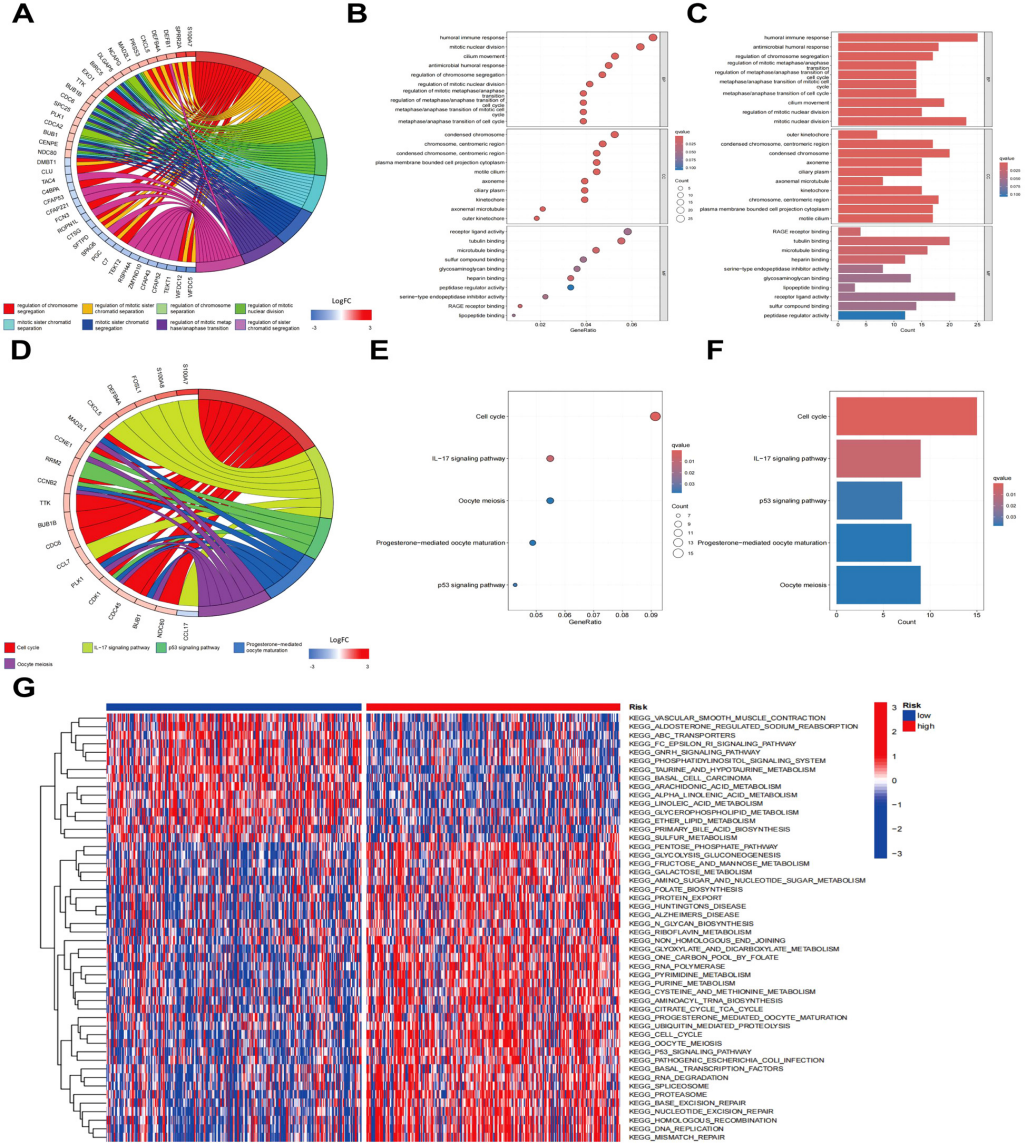
Enrichment analysis of risk genes. **(A)** Clustering diagram of GO enrichment analysis. **(B)** Histogram of GO enrichment analysis. **(C)** Bubble plot of GO enrichment analysis. **(D)** Clustering diagram of KEGG enrichment analysis. **(E)** Bar chart of KEGG pathway enrichment analysis. **(F)** Bubble plot of KEGG pathway enrichment analysis. **(G)** Heatmap of GSVA enrichment analysis.

To clarify the gene expression differences between the high - risk and low - risk groups and gain a deeper understanding of the molecular mechanisms of LUAD. Differential gene expression analysis between the high-risk and low-risk cohorts was executed, leading to the identification of 473 risk-related genes. The top 50 genes exhibiting the greatest |log2FC| values were chosen for CA, as depicted in the heatmap in **Supplementary Figure S1B**. To investigate the possible biological functions of these risk-related genes in LUAD, functional enrichment evaluation was executed utilizing GO and KEGG pathway analyses on all identified risk genes (**Figures 5A–F**). The findings indicated that pathways linked to the “humoral immune response”, “mitotic nuclear division”, and “cell cycle” were markedly enriched in the high-risk cohort. In addition, to assess the variations in pathway activity between the risk cohorts, gene set variation analysis (GSVA) was executed, with the findings presented in **Figure 5G**. Pathways encompassing “cell cycle”, “nucleotide excision repair”, “proteasome”, and “pyrimidine metabolism” demonstrated notably higher activity in the high-risk cohort relative to the low-risk cohort (*P* < 0.05). These observations suggest that risk-associated genes are predominantly implicated in biological mechanisms associated with the cell cycle.

### 3.6 Development of PPI network

**Figure 6:**
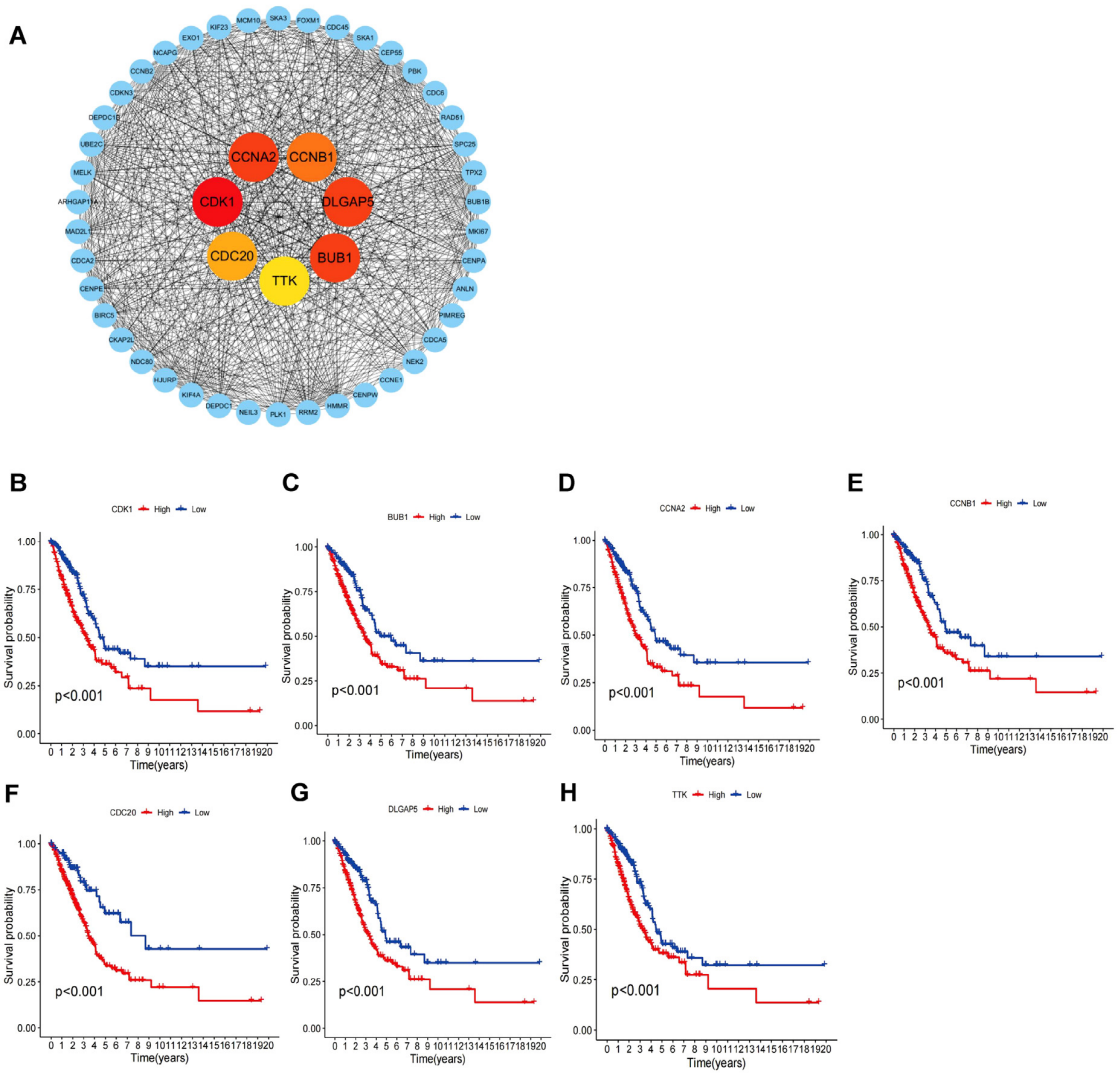
PPI network and prognostic value of core genes. **(A)** PPI network diagram, where darker colors indicate stronger correlations with other genes. **(B-H)** Kaplan-Meier plots for CDK1, BUB1, CCNA2, CCNB1, CDC20, DLGAP5, and TTK in high-risk and low-risk patient cohorts.

To clarify the gene expression differences between the high - risk and low - risk groups and gain a deeper understanding of the molecular mechanisms of LUAD. The PPI network was constructed employing the Strings database (**Supplementary Figure S7A**). Subsequently, the PPI network was visualized through Cytoscape software (**Supplementary Figure S7B**). The top 7 genes with the highest degree of correlation were identified employing the ‘cytohubba’ algorithm, a plugin within Cytoscape. As depicted in **Figure 6A**, CDK1 showed the strongest correlation with risk genes, followed by DLGAP5, CCNA2, BUB1, CCNB1, TTK, and CDC20. Additionally, robust associations were identified among these seven genes. To further evaluate the prognostic significance of these genes, a Kaplan-Meier analysis was conducted. The findings indicated that elevated expression levels of CDK1, DLGAP5, CCNA2, BUB1, CCNB1, TTK, and CDC20 were markedly linked to poorer OS (*P* < 0.05), as illustrated in **Figures 6B–H**.

### 3.7 Key genes validation

**Figure 7:**
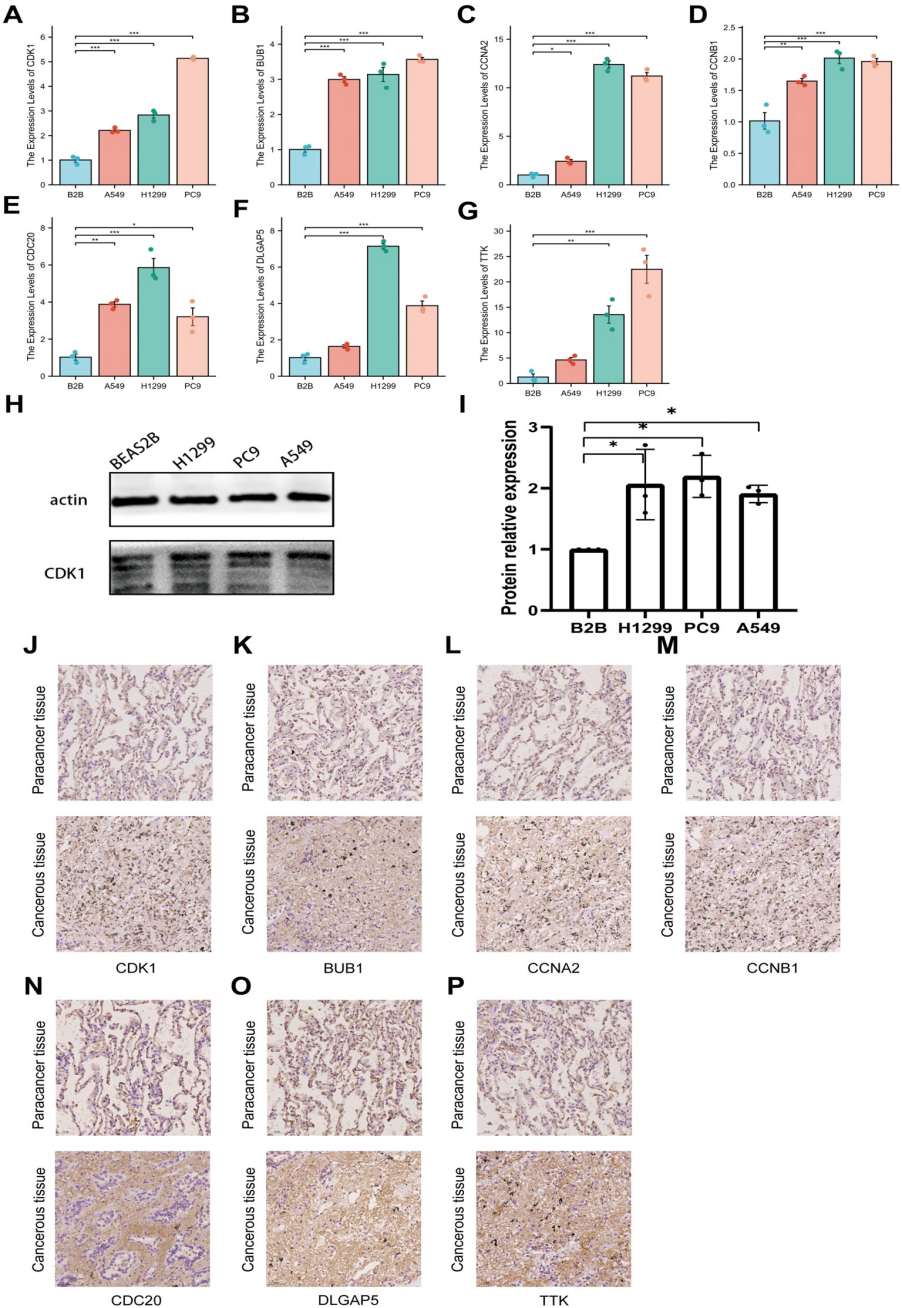
Expression profiles of seven genes in different CLs. **(A–G)** Relative expression levels of CDK1, BUB1, CCNA2, CCNB1, CDC20, DLGAP5, and TTK in LUAD cells and normal cells were validated by PCR. **(H, I)** Relative expression levels of CDK1 in LUAD cells and normal cells were validated by WB. **(J–P)** Immunohistochemical staining of CDK1, BUB1, CCNA2, CCNB1, CDC20, DLGAP5, and TTK in clinical samples, with images magnified 100x. *P<0.05, **P<0.01, ***P<0.005.

To verify the differential expression of the seven target genes between LUAD and normal lung cells, qRT-PCR analysis was carried out. The findings revealed a substantial elevate in the expression levels of CDK1, BUB1, CCNA2, CCNB1, TTK, CDC20, and DLGAP5 in A549, PC9, and H1299 CLs, in comparison to the Beas-2B CL (**Figures 7A–G**). Moreover, WB analysis was employed to assess CDK1, the gene exhibiting the strongest correlation (**Figures 7H, I**). It was observed that CDK1 expression was considerably higher in the three LUAD CLs relative to Beas-2B cells. To explore whether similar expression patterns are present *in vivo*, immunohistochemistry (IHC) staining was executed on tumor tissues and adjacent normal tissues from LUAD patients. The results illustrated a notable overexpression of CDK1, BUB1, CCNA2, CCNB1, CDC20, DLGAP5, and TTK in LUAD tissues (**Figures 7J–P**).

### 3.8 CA between RS model and immunotherapy

**Figure 8:**
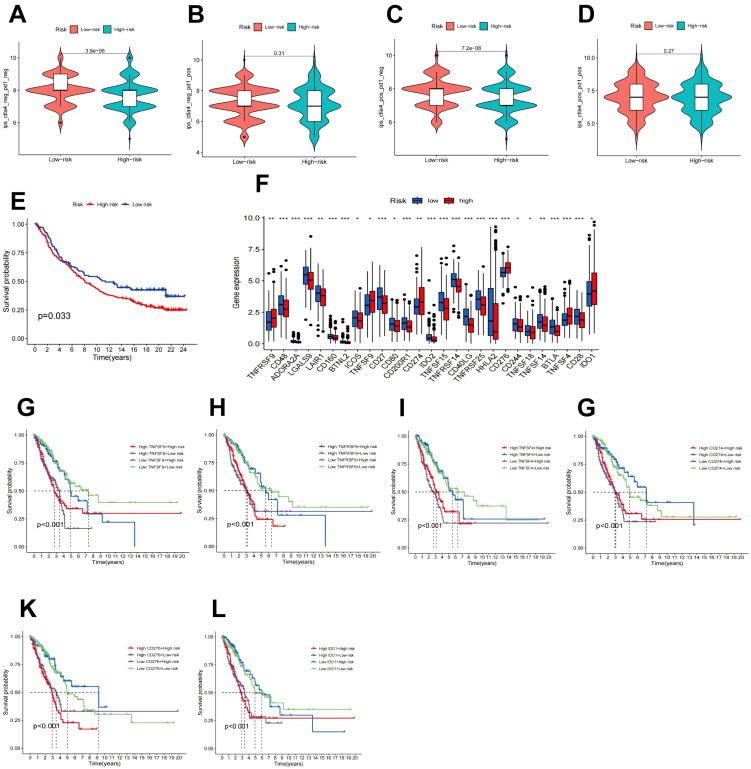
Immune checkpoint analysis and prediction of immunotherapy response. **(A–D)** The impact of CTLA4/PD1 drug usage on IPS in high-risk and low-risk patients. **(E)** Kaplan-Meier survival analysis of the IMvigor210 treatment cohort. **(F)** Expression of immune checkpoint genes in high-risk and low-risk populations. **(G–L)** Kaplan-Meier survival curves for OS of four patient cohorts categorized by RS and expression levels of TNFRSF9, TNFSF9, TNFSF4, CD274, CD276, or IDO1.

Immunotherapy is considered a vital therapeutic strategy for patients with LUAD. Recent studies suggest that FAM may modulate tumor biological behavior by affecting the local TME (22). Consequently, the relationship between the RS model derived from FAM genes and the effectiveness of immune checkpoint inhibitors (ICIs) was examined. First, the IC50 values for multiple anticancer agents were predicted for each sample, and the variations between high-risk and low-risk cohorts were assessed. The outcomes indicated that the IC50 values for most drugs (such as axitinib, BMS, doramapimod, and ribociclib) were markedly lower in the low-risk cohort relative to the high-risk cohort, as illustrated in **Supplementary Figure S8**. Further analysis of the TCIA database (**Figures 8A–D**) revealed that the low-risk cohort displayed notably higher immunotherapy scores (IPS) and responses to anti-CTLA4 treatment (*P* < 0.001), implying that individuals with lower RS may experience enhanced benefits from ICIs. To substantiate the predictive capacity of the RS in the context of immunotherapy, a Kaplan-Meier survival analysis was conducted employing data from the IMvigor210 immunotherapy cohort. The findings confirmed that within the IMvigor210 cohort, lower RS correlated with improved OS in immunotherapy (**Figure 8E**).

The abundance of immune checkpoint genes between the high-risk and low-risk patient cohorts was further evaluated. As depicted in **Figure 8F**, 27 genes exhibited differential expression patterns. In contrast to the low-risk cohort, individuals in the high-risk cohort displayed elevated levels of TNFRSF9, TNFSF9, TNFSF4, CD274, CD276, and IDO1. Furthermore, the transcript abundance of these genes demonstrated a negative association with OS (**Figures 8G-L**).

Single-gene immune cell CA was executed for the seven key genes included in the RS model, as presented in **Supplementary Figure S9**. All seven genes showed positive correlations to CD^8+^ T cells, CD^4+^ T cells, and regulatory T cells, while exhibiting negative links to B cells, monocytes, dendritic cells, and mast cells.

These findings suggest that FAM-related genes could modulate the TME by influencing immune cell infiltration and the expression of immune checkpoint-related genes, thereby impacting the effectiveness of immunotherapy.

## 4 Discussion

This study identified key genes through a prognostic model utilizing FAM-related genes, elucidated the significant roles of these seven genes in LUAD, and explored the impact of FAM on immunotherapy, providing novel scientific evidence for LUAD treatment. Cancer has become one of the most urgent worldwide public health challenges, with LC being the leading cause of mortality (23). Moreover, LUAD represents the most prevalent subtype of LC (24). Despite advances in LUAD therapies, the development of effective prognostic markers and molecularly targeted treatments remains limited (25). A hallmark of cancer is abnormal cell growth and proliferation, which necessitates large quantities of protoplasmic components such as nucleic acids, proteins, and lipids for its initiation and progression (26). FAM plays a pivotal role in the metabolic transformation of nutrients, which has drawn attention to its potential significance in tumor therapy (27–29). Nonetheless, research on FAM in LUAD remains scarce. In this study, 125 FAM genes linked to cancer progression were detected using the TCGA-LUAD dataset. A prognostic model was developed via LASSO regression and cross-validation, demonstrating consistent predictive capability across multiple datasets. Survival analysis demonstrated that LUAD patients with higher RS had a poorer prognosis. Clinical CA revealed that the RS was markedly associated with gender, N stage, clinical stage, and T stage. Furthermore, univariate and multivariate Cox analyses suggested that the RS could function as an independent prognostic marker for LUAD. Additionally, the RS exhibited a positive correlated to the mRNAsi index of CSCs, further underscoring its role in prognostic assessment.

In recent years, the influence of cytokines on cancer has attracted significant attention, with profound implications for both clinical and translational medical research (30). Cytokines can affect cancer progression, metastasis, and the immune microenvironment through various pathways. For example, the interleukin (IL) family promotes tumor development by stimulating tumor cell proliferation, growth, and angiogenesis (31), whereas IFNs inhibit tumor progression by restraining cancer cell proliferation, inducing apoptosis, and modulating immune responses. Accordingly, the link between RS and cytokine expression was investigated, revealing notable variations in the levels of multiple cytokines among high- and low-risk cohorts. The results indicated that CCL8, CCL20, CXCL10, IFNGR2, TGFBR1, and VEGFBR1 were positively correlated to RS, while CS3CR1, IL-10, TGFBR, and IL-12B exhibited negative associations. CCL8, CCL20, and CXCL10 are key players in regulating immune cell migration and inflammatory responses, contributing essential roles in immune surveillance and TME modulation (32, 33). IFNGR2 and VEGFBR1 also impact tumor progression by modulating immune recognition, nutrient supply, and the metastatic potential of tumor cells (34, 35). IL-10, one of the earliest identified cytokines, has the ability to suppress cancer development and metastasis by modulating inflammation and immune responses (36). TGFBR is known to function as a cancer inhibitor due to its ability to inhibit epithelial cell proliferation; nevertheless, in advanced stages of the disease, TGFBR seems to facilitate tumor advancement (37). In conclusion, these cytokines and receptors serve crucial functions in governing the TME, modulating immune responses, and serving as potential therapeutic targets. Further research into their regulatory mechanisms and interactions may offer new therapeutic strategies and opportunities for personalized treatment.

Finally, a nomogram was developed utilizing factors like RS, age, gender, and disease stage to predict patient survival, demonstrating a high level of concordance with observed survival outcomes. Differential risk analysis was executed using the prognostic model, identifying 473 DEGs associated with risk. Subsequent functional analysis of these genes suggested that in the high-risk cohort, we discovered that high-risk genes were significantly enriched in pathways associated with humoral immune response and the cell cycle. Previous studies have shown that NME4, a potential immunotherapeutic target, promotes immune evasion in LUAD by suppressing CD8+ T cell infiltration, and its overexpression is strongly correlated with enhanced cellular proliferation, increased metastatic potential, and poor clinical outcomes (38). These findings are consistent with our results. Tumor cells, through surface-expressed abnormal antigens, induce B cells to produce ineffective antibodies and activate negative regulatory signals in B cells, resulting in the failure of immune surveillance. Moreover, the B-cell receptor signaling pathway is abnormal. Tumor cells aberrant signaling nodes, prompting B cells to secrete immunosuppressive factors, ultimately leading to immune escape and creating an inflammatory microenvironment conducive to tumor growth and metastasis. Regarding the cell-cycle-related pathways, gene sets associated with cycle regulation, DNA replication, etc. are highly enriched, manifested as the dysregulation of pathways such as Rb-E2F and the abnormal activation of the CDK-cyclin complex. This leads to uncontrolled cell proliferation and genomic instability. Given the central role these key pathways play in the pathogenesis of LUAD, developing drugs to block the abnormal interactions between tumor cells and B cells or to regulate key molecules for the humoral immune response pathways, as well as developing CDK inhibitors or restoring the Rb-E2F pathway function through gene therapy for the cell-cycle-related pathways, represents promising therapeutic strategies for LUAD treatment.

Subsequently, seven genes (CDK1, DLGAP5, CCNA2, BUB1, CCNB1, TTK, and CDC20) most strongly associated with risk genes were selected for detailed analysis, revealing their substantial impact on LUAD patients survival. PCR and WB experiments verified that the expression levels of these seven crucial genes were notably elevated in LUAD CLs relative to normal lung cells. Furthermore, IHC confirmed their elevated expression in tumor tissues of LUAD patients in contrast to normal tissues. Importantly, these seven key genes have been identified as positive regulators of the cell cycle in numerous cancer studies, playing essential roles in cancer development (39–45). CDK1, the only essential cyclin-dependent kinase in human cells, is vital for cell growth, proliferation, and replication (46). It also acts as an upstream regulator for BUB1, CCNB, and CDC20 (47–49). Additionally, CDK1 promotes tumor development and enhances drug resistance by influencing cell morphology, motility, and apoptosis (39, 50, 51). It has been reported that CDK1 can bind directly and phosphorylate acyl-CoA synthetase long-chain family member 4 at the S447 site, thereby blocking the biosynthesis of lipid-containing polyunsaturated fatty acids, inhibiting lipid peroxidation, and preventing ferroptosis (50). Recent research has demonstrated that CDK1 is over expressed in cancer and is intimately associated with unfavorable outcomes in LUAD (52). DLG-associated protein 5 (DLGAP5) has been recognized as a cell cycle regulatory protein (53) that enhances k-fiber stability and facilitates chromosome alignment by influencing Kif18A localization and dynamics at kinetochore microtubule plus ends (54). DLGAP5 is strongly correlated with cancer occurrence and poor prognosis across multiple cancer types (55). In osteosarcoma, DLGAP5 has been demonstrated to promote tumor development via the IL-6/JAK2/STAT3 signaling pathway (56). CCNA2 is a well-known cell proliferation regulator and has been employed as a proliferation marker for molecular diagnostics (57). Several investigations have demonstrated that CCNA2 can alter cell proliferation and differentiation by affecting various pathways, promoting tumor formation and progression and contributing to adverse pathological outcomes (41, 58, 59). BUB1 is a crucial protein-coding gene involved in spindle assembly checkpoint signaling and proper chromosome alignment (60). NingJiang et al. found that BUB1 kinase promotes the advancement and multiplication of human bladder cancer (BCa) by modulating the transcriptional activation of STAT3 signaling, making it a potential target for BCa therapy (61). However, fewer studies have explored the role of BUB1 in LUAD, warranting further research. CCNB1, TTK, and CDC20 are recognized regulators of the cell cycle, ensuring proper chromosome segregation during mitosis (62). Current studies suggest that CCNB1, TTK, and CDC20 are overexpressed in various cancers (63–65). These seven key genes are intimately linked to the cell cycle, indicating a strong association between FAM alterations and cell cycle dysregulation in LUAD. CDK1 has been shown to activate Pah1 and Tgl4, promoting lipolysis and providing fatty acids necessary for the completion of the late G1 phase of the cell cycle (66). DLGAP5 and BUB1 exert regulatory effects on STAT3 (67), which can bind to the FASN promoter region and promote *de novo* fatty acid synthesis (67). CCNB1 is capable of stimulating fatty acid oxidation to meet the increased metabolic demands of cancer cells by modulating the p53 pathway (68). Furthermore, numerous studies have revealed that tumor cells undergo metabolic reprogramming to meet their heightened demands for rapid proliferation and survival, a process that is significantly correlated with patient prognosis (69, 70). Thus, restoring the normal FAM environment in the body is of significant importance for restraining the abnormal proliferation of LUAD cells and impeding the progression of LUAD. Furthermore, dysregulation of fatty acid metabolism is closely associated with the prognosis of LUAD patients, underscoring the potential clinical value of modulating fatty acid metabolic pathways to improve patient outcomes.

Immunotherapy, as an innovative approach to cancer treatment, has garnered increasing attention in the clinical application of LUAD management (71). Recent studies have shown that FAM can modulate the tumor immune microenvironment (72, 73), and enrichment analysis results indicate that genes within the high-risk cohort are primarily linked to immune pathways. Consequently, drug sensitivity analysis reveals that medications like axitinib, BMS, doramapimod, and ribociclib exhibit markedly improved efficacy in the low-risk cohort relative to the high-risk cohort. Findings from immunotherapy analysis suggest that low-risk patients tend to respond more favorably to ICIs. Validation through the IMvigor210 cohort corroborates this perspective, underscoring the potential utility of the FAM prognostic model to guide immunotherapy in LUAD. Subsequent examination comparing immune checkpoint gene expression between high- and low-risk cohorts indicated elevated expression of TNFRSF9, TNFSF9, TNFSF4, CD274, CD276, and IDO1 in high-risk patients, potentially affecting immunotherapy effectiveness and prognosis, and offering valuable insights for personalized therapeutic strategies. Given the significant role of immune checkpoints on the immune microenvironment, single-gene immune cell CA was conducted on seven pivotal genes, revealing their potential influence on tumor immune cell infiltration. In this investigation, the expression of all seven genes was positively correlated with CD^8+^ T cells, CD^4+^ T cells, and regulatory T cell infiltration, while being negatively associated with B cells, monocytes, dendritic cells, and mast cell infiltration. B cells serve various functions within the human body, encompassing antigen presentation and antibody production, supporting T cell responses and related complement, macrophage, and natural killer cell mechanisms, all of which possess significant prognostic relevance (74). Current studies have shown that the synthesis of monounsaturated fatty acids directly governs B cell differentiation (75). Additionally, germinal center B cells, known for their high proliferation, do not rely on glycolysis for energy but sustain their energy requirements through fatty acid oxidation (76). Research on prostate cancer has demonstrated that the depletion of CDC20 can enhance CD8 T lymphocyte infiltration in a GSDME-dependent manner, thereby amplifying antitumor immunity (77). This study elucidates the pivotal role of seven key genes in tumor immune infiltration, particularly regarding their impact on T cells and B cells, and underscores the significance of FAM alterations. Thus, the FAM RS model could potentially influence the efficacy of immunotherapy by regulating immune checkpoint gene expression and immune cell infiltration.

Unlike traditional prognostic models that are primarily relying on clinicopathological features and thus insufficiently consider the molecular characteristics of tumors, as well as molecular prognostic models that focus on a single signaling pathway or a certain category of genes, the prognostic model we constructed based on FAM genes commences from FAM-related genes. It delves into the correlation between the tumor-intrinsic metabolic dysregulation of tumors and prognosis, providing a novel perspective. Moreover, it comprehensively takes into account multiple genes that are closely associated with fatty acid metabolism and the cell cycle. Its predictive stability and accuracy have been validated across different datasets, allowing it to more accurately reflect the biological behavior of tumors. Furthermore, this model holds great significance for treatment decision-making. For patients with a high-risk score, which indicates a high degree of tumor malignancy and poor prognosis, clinicians can adopt proactive strategies such as intensified chemotherapy regimens, early-stage immunotherapy, or combined targeted therapy to improve patient survival outcomes. In contrast, for patients with a low-risk score, the treatment intensity can be appropriately reduced on the premise of ensuring treatment efficacy, so as to minimize unnecessary treatment - related side effects and improve the patients’ quality of life. The key genes screened by the model also offer potential targets for the development of targeted drugs. In the future, further prospective clinical studies will help verify its effectiveness and reliability, thereby accelerating the translation of basic research findings into clinical applications.

In conclusion, a prognostic model utilizing FAM genes has been developed. The reliability and precision of this model were confirmed through survival analysis and further validated in additional datasets. When combined with patients’ clinical profiles, it exhibited its significance as an independent prognostic factor for predicting outcomes in LUAD patients. Furthermore, a nomogram that integrates RS with several critical clinical parameters was developed, offering a valuable tool for prognostic evaluation in LUAD. Key genes were identified through differential gene analysis employing RS. The clinical relevance and expression profiles of seven key genes in LUAD patients were validated through survival analysis and *in vitro* experiments. Additionally, the associations between RS and the efficacy of immunotherapy, expression of immune checkpoint-related genes, and immune cell infiltration were investigated. In summary, this study provides robust scientific evidence for exploring treatment strategies for LUAD and establishes a foundation for future translational clinical research.

## Data Availability

The original contributions presented in the study are included in the article/**Supplementary Material**. Further inquiries can be directed to the corresponding authors.
