# Supplementary material for: Fatty acid metabolism-derived prognostic model for lung adenocarcinoma: unraveling the link to survival and immune response
Source: Front Immunol. 2025 Mar 13;16:1507845. doi: 10.3389/fimmu.2025.1507845 (PMC11965909; doi:10.3389/fimmu.2025.1507845)
Supplement: Supplementary file 1 [file DataSheet1.pdf]

CCNB1	ATACTGCCTCTCCAAGCCCAATG	23
	AGCTCCATCTTCTGCATCCACATC	24
CCNA2	ATTGCTGGAGCTGCCTTTCATTTAG	25
	TGCTGTGGTGCTTTGAGGTAGG	22
TTK	TTCCAGCAGCAACAGCATCAAATAC	25
	TGCTTGAACCTCCACTTCCTATCTG	25
BUB1	CCATGAGGATCTGCCCGCTTC	21
	ACTGGTGTCTGCTGATAGGTTACTG	25
CDK1	AAGGGGTTCCTAGTACTGCAATTCG	25
	GCATAAGCACATCCTGAAGACTGAC	25
CDC20	GCAGACATTCACCCAGCATCAAG	23
	CATCCACGGCACTCAGACAGG	21
DLGAP5	CCCAGAACAGTCTCATCTACCACAG	25
	GTCTTCCTTTACTTGGCACCTTTCC	25

Supplementary Table 1. Primer sequence and number of base pairs for verified gene expression

Gene	Coef
ACSBG1	-0.319081066
HACD1	0.156690125
ELOVL2	0.130546288
ENO3	-0.12775706
CEL	-0.028314992
ACOXL	-0.027831353
LDHA	0.236880211
ACSL1	-0.006014941
ALOX5AP	-0.072578146
SMS	0.114601293
ALOX12B	0.156790879
ALDOA	0.100055966
CYP4B1	-0.049899076
DPEP2	-0.137275494
ELOVL6	0.017331862
HSD17B7	-0.087810137

Supplementary Table 2. The formula of Coef composition of each gene was obtained according to the model.

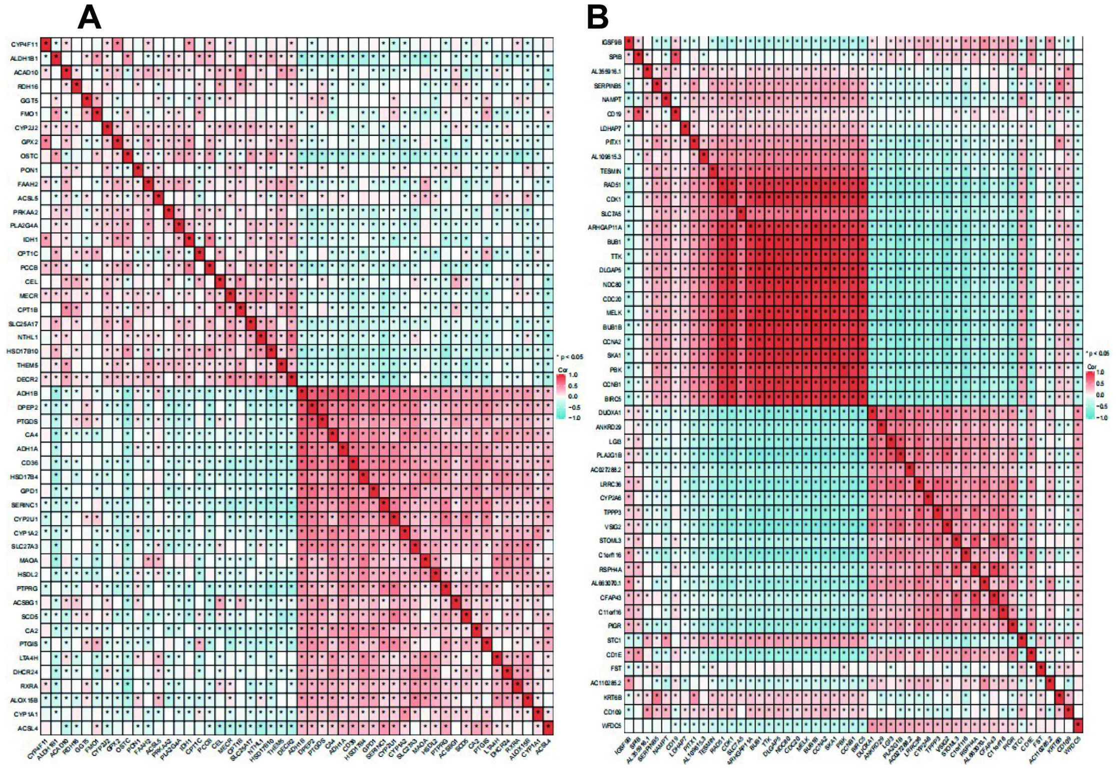

Figure S1. Correlation analysis of genes related to fatty acid metabolism. (A)|log2FC| top 50 fatty acid metabolism gene correlation heat maps. (B)|log2FC| heat maps of the top 50 risk gene associations.

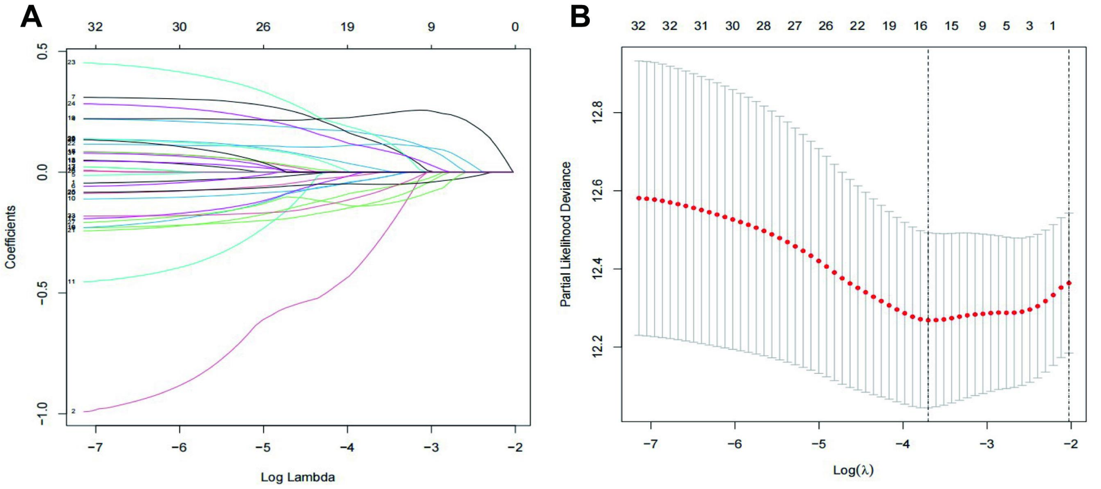

Figure S2. Risk model of LUAD patients based on fatty acid-related genes (A) Select tuning parameters (log l) for OS-related proteins to cross-validate error curves. According to the minimum criterion and the 1-se criterion, draw a vertical dotted line at the optimal value. (B) Plot LASSO coefficient distributions for 30 OS-associated lncrnas and draw vertical dashed lines at the values selected for 10x cross-validation.

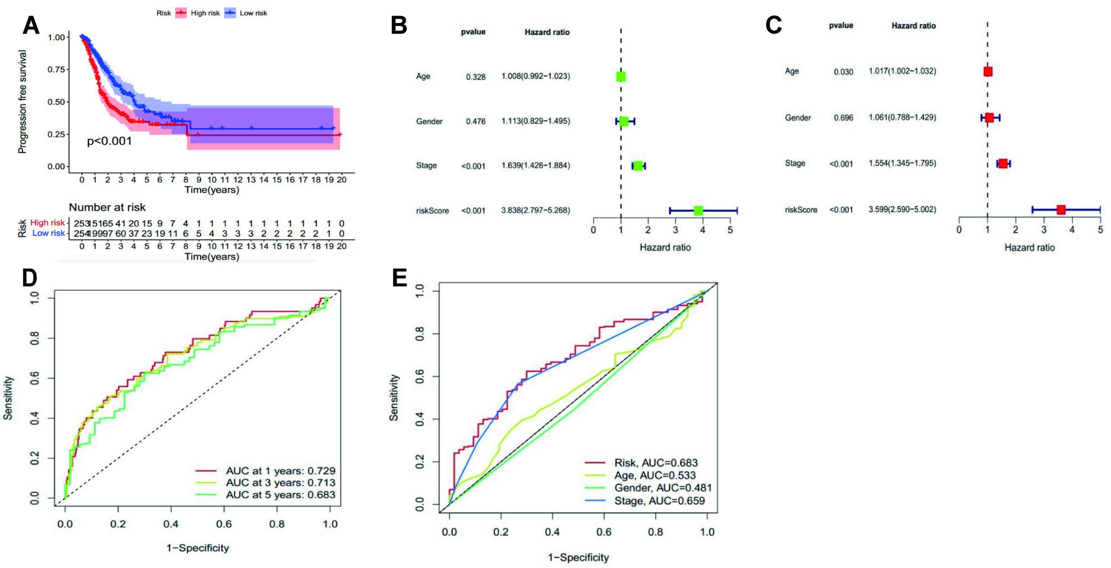

Figure S3. The prognostic risk model evaluated the RS and clinical features of LUAD in the TCGA group (A) Univariate analysis of clinical features with RS and OS. (B) Multifactor analysis ofclinical features with RS and OS. (C) ROC curves of RS and predicted survival time of 1, 3, and 5 years. (D) ROC curve of clinical features and RS.

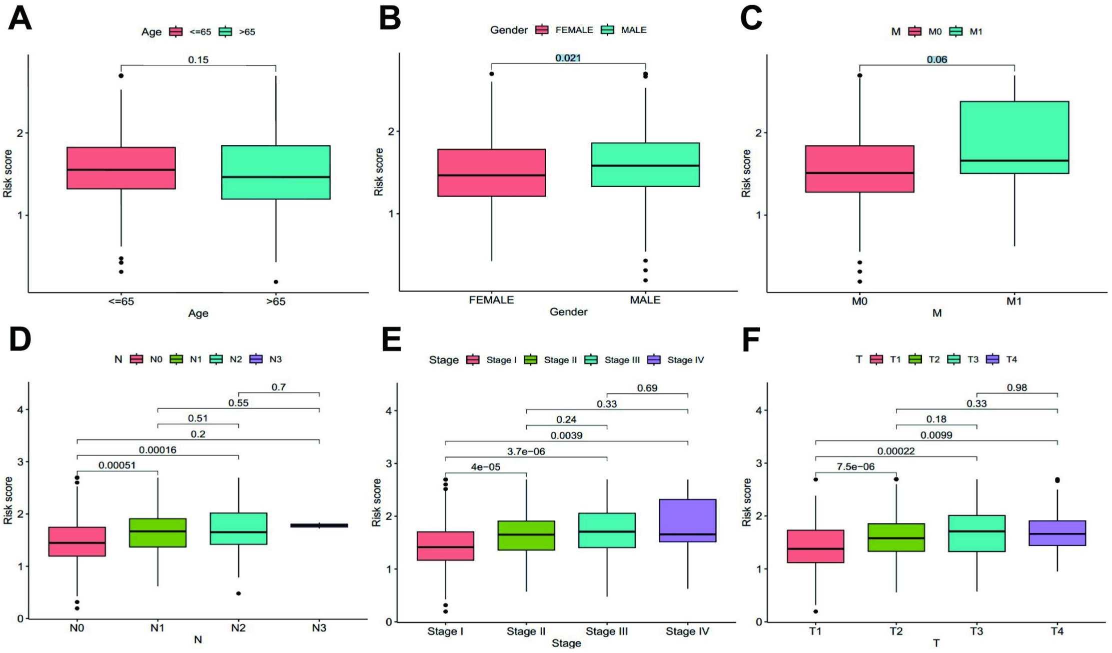

Figure S4. Clinical correlation analysis of LUAD patients (A-F) analyzed OS differences between high-risk and low-risk groups in the TCGA package: sex, age, tumor grade, or TNM stage.

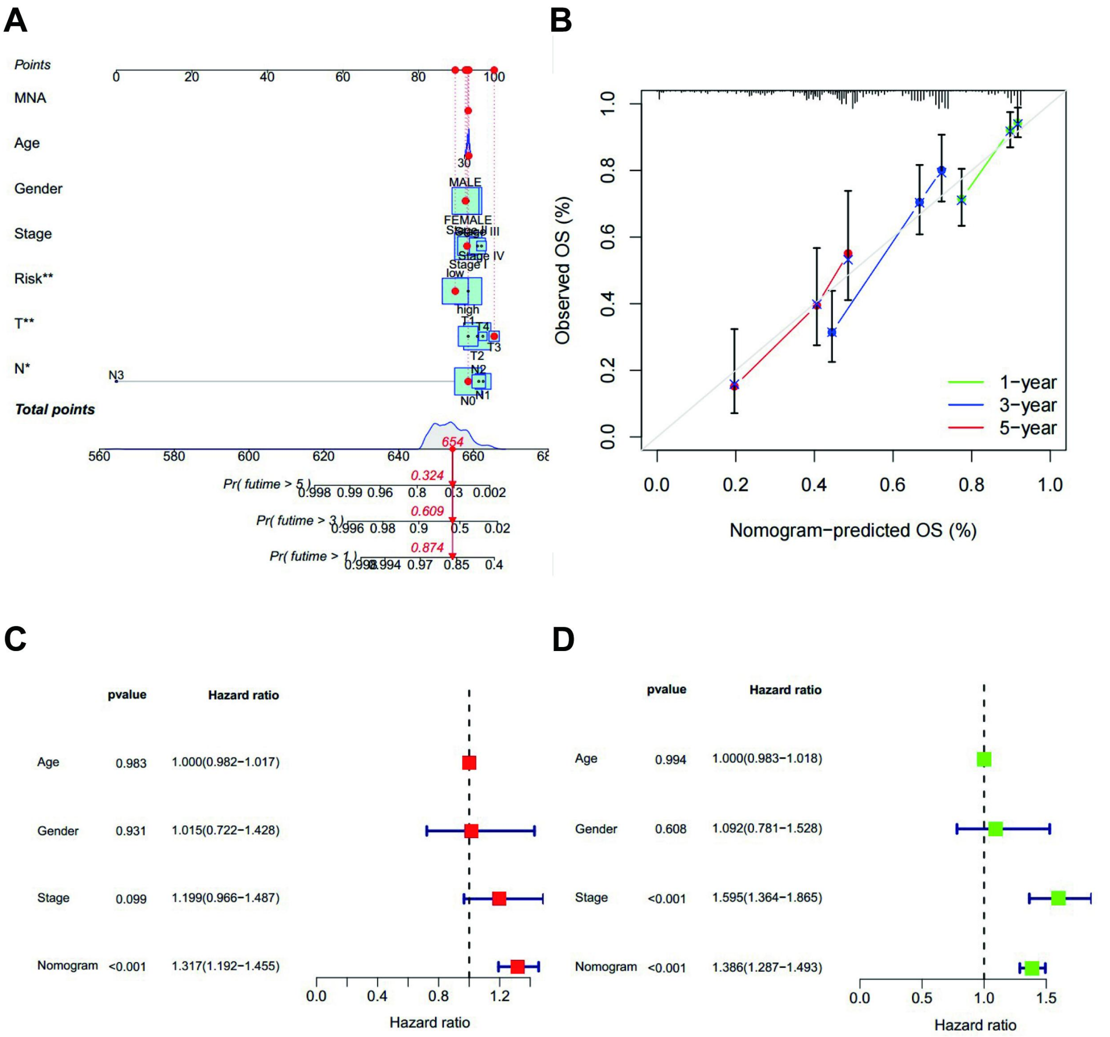

Figure S5. A nomogram based on risk scores and clinical factors (A) Nomograms of 1 , 3 , and 5-year survival rates were analyzed. (B) Calibration curves for 1, 3, and 5-year survival rates based on the nomogram. (C) Monofactorial analysis of the monogram with other influencing factors and OS. (D) Multi-factor analysis of the monogram with other influencing factors and OS.

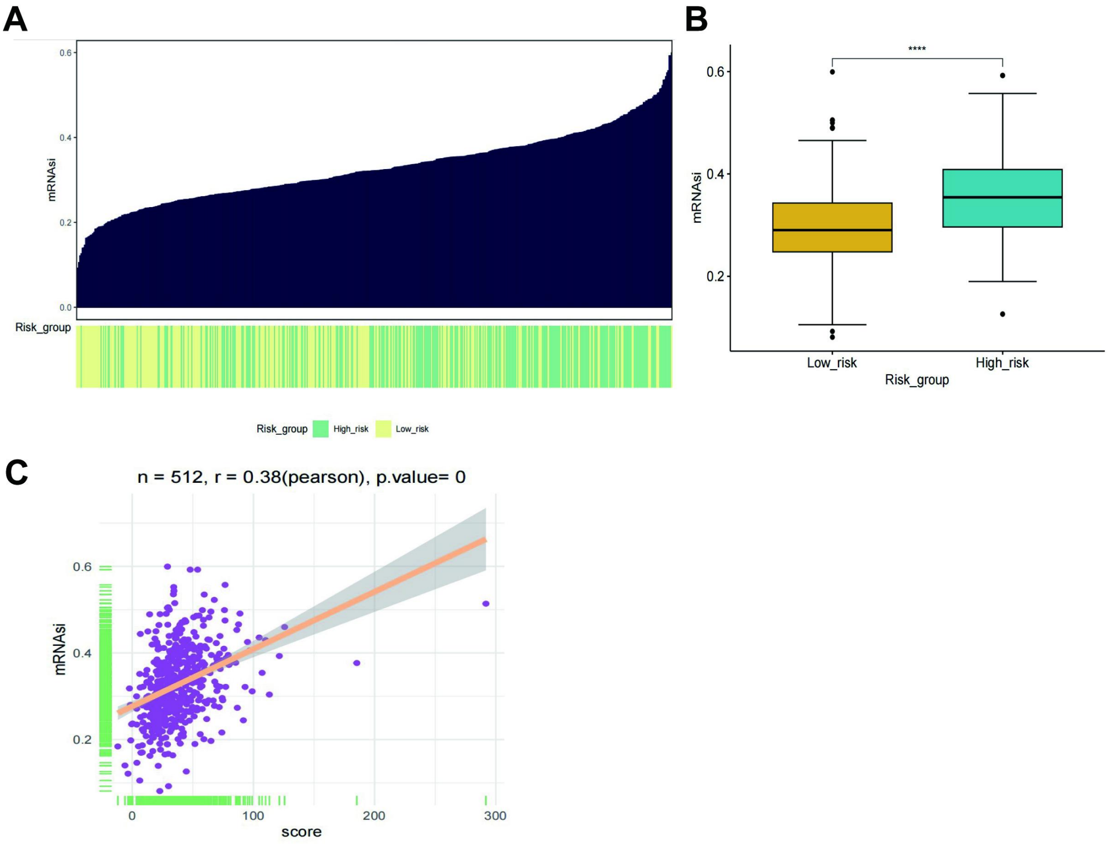

Figure S6. Tumor stem cell analysis of LUAD patients (A) Correlation point plots of mRNAsi scores for each patient divided into high and low risk groups based on RS (B) differences in mRNAsi scores between different groups (C) Correlation analysis plots between RS and mRNAsi scores, with trend lines.

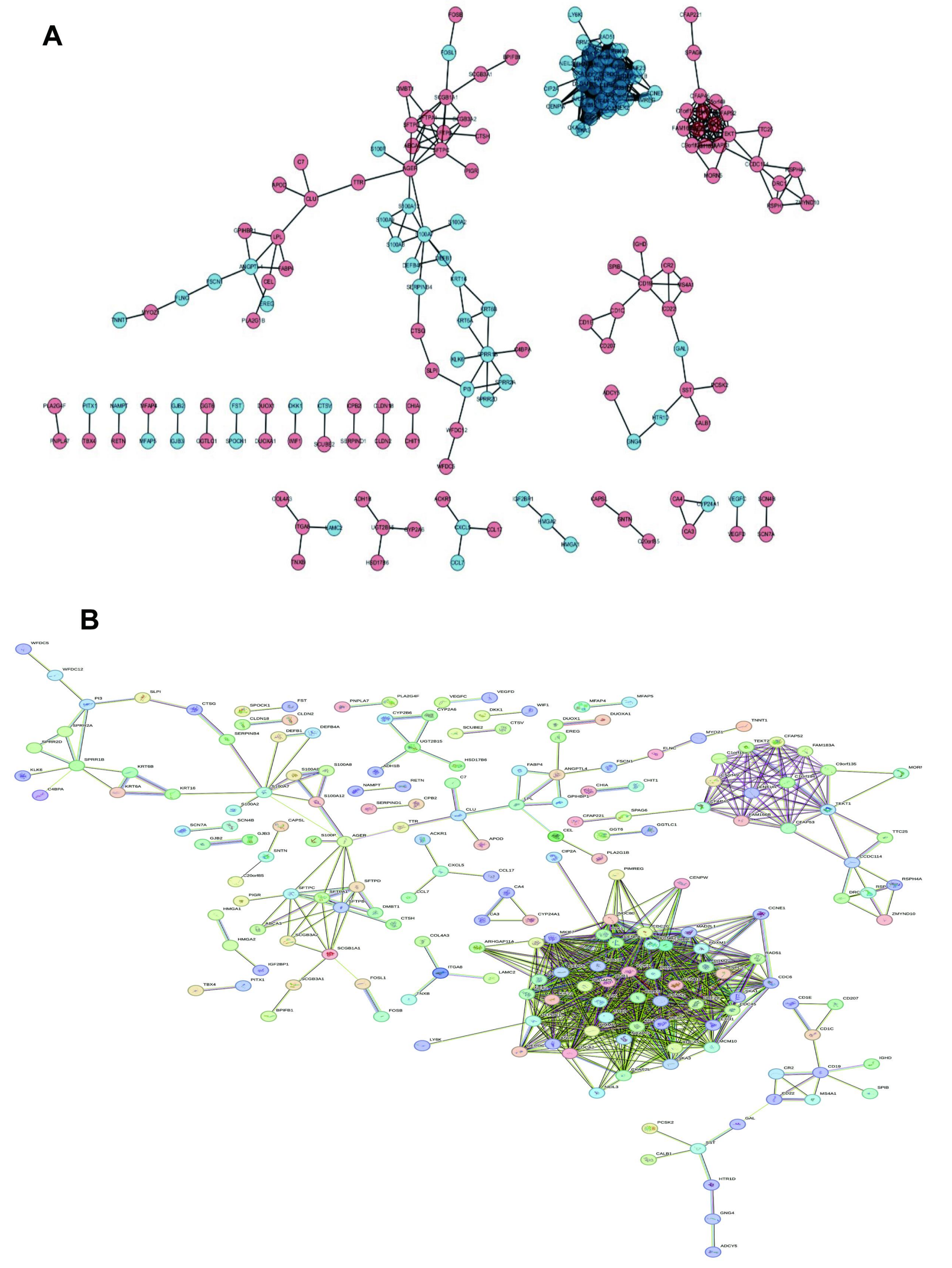

Figure S7. Protein interaction network (A) Protein interaction network diagram drawn by String. (B)Cytoscape software visualizes protein interaction network schematics.

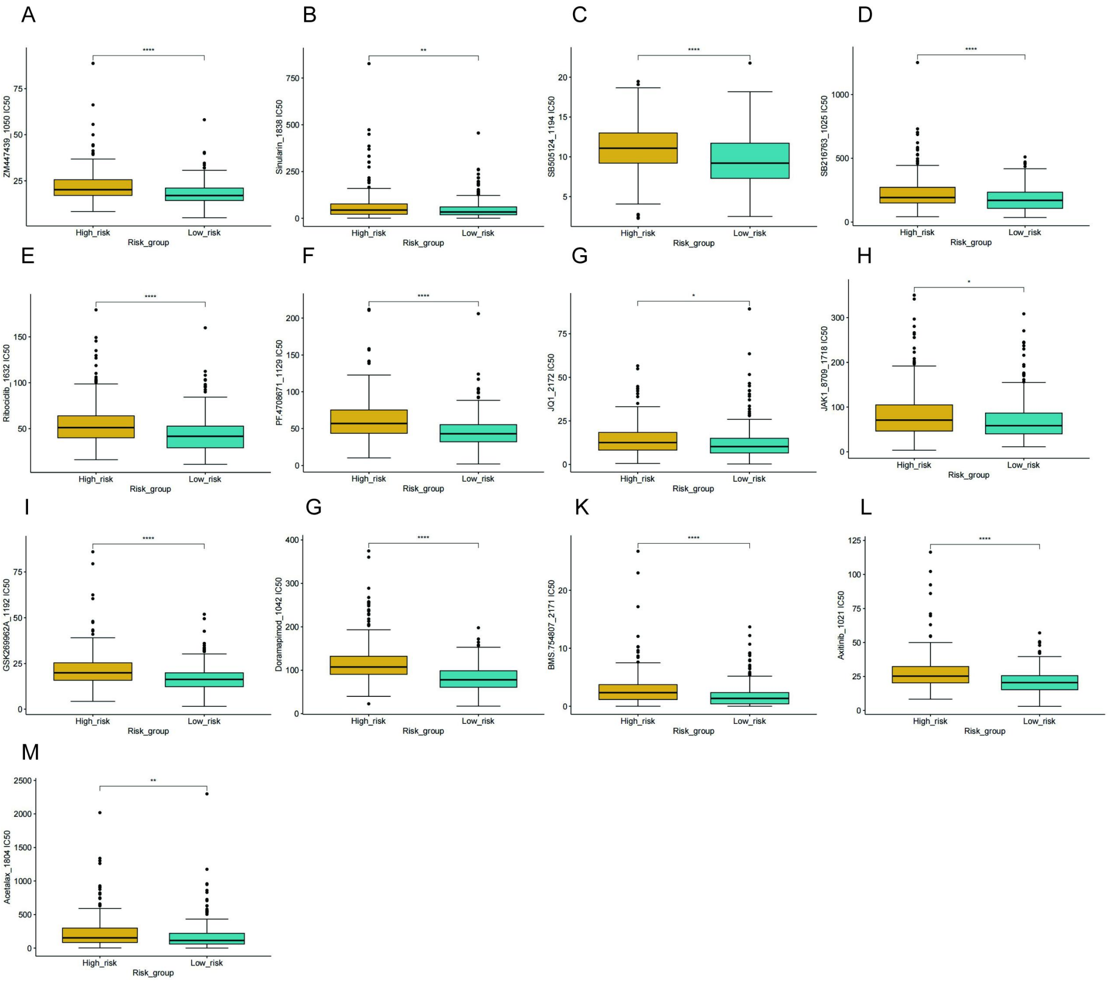

Figure S8. Drug sensitivity analysis diagram. (A-M) Box plot of IC50 differences between LUAD patients for different chemotherapy agents between high and low risk groups.

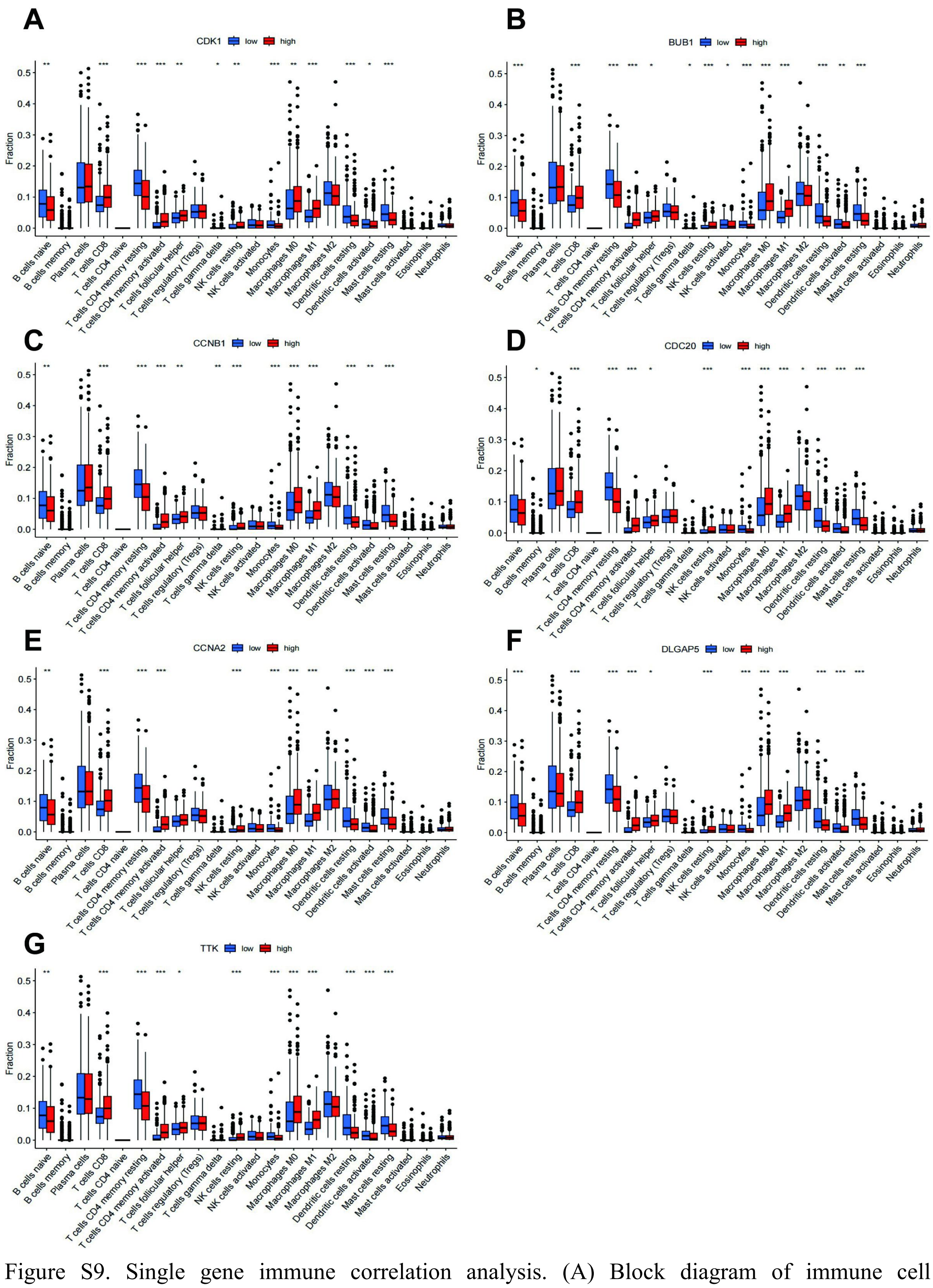

Figure S9. Single gene immune correlation analysis. (A) Block diagram of immune cell expression in CDK1 low-risk and high-risk groups. (B) Block diagram of immune cell expression in low-risk and high-risk groups BUB1B. (C) Block diagram of immune cell expression in low-risk and high-risk CCNA2 groups. (D) Block diagram of immune cell expression in low-risk and high-risk CCNB1 groups. (E) Block diagram of immune cell expression in CDC20 low-risk and high-risk groups. (F) Block diagram of immune cell expression in DLGAP5 low-risk and high-risk groups. (G) Block diagram of immune cell expression in low-risk and high-risk TTK groups.
