# Supplementary material for: Fatty acid metabolism-derived prognostic model for lung adenocarcinoma: unraveling the link to survival and immune response
Source: Front Immunol. 2025 Mar 13;16:1507845. doi: 10.3389/fimmu.2025.1507845 (PMC11965909; doi:10.3389/fimmu.2025.1507845)
Supplement: Supplementary file 2 [file DataSheet2.zip › Data analysis/worksheet.docx]

Download the TCGA-LUAD dataset and the GEO dataset GSE68465 and collate them using 'merge.pl', 'moveMutFiles.pl', 'getClinical.pl' and 'probe2symbol.pl', TMB via 'TMb.pl'. Download the fatty acid metabolism gene "gene.txt" from MsigDB, <https://www.gsea-msigdb.org/gsea/msigdb/.> All subsequent analysis methods are presented using R files
