# Supplementary figures and images for: Fatty acid metabolism-derived prognostic model for lung adenocarcinoma: unraveling the link to survival and immune response
Source: Front Immunol. 2025 Mar 13;16:1507845. doi: 10.3389/fimmu.2025.1507845 (PMC11965909; doi:10.3389/fimmu.2025.1507845)

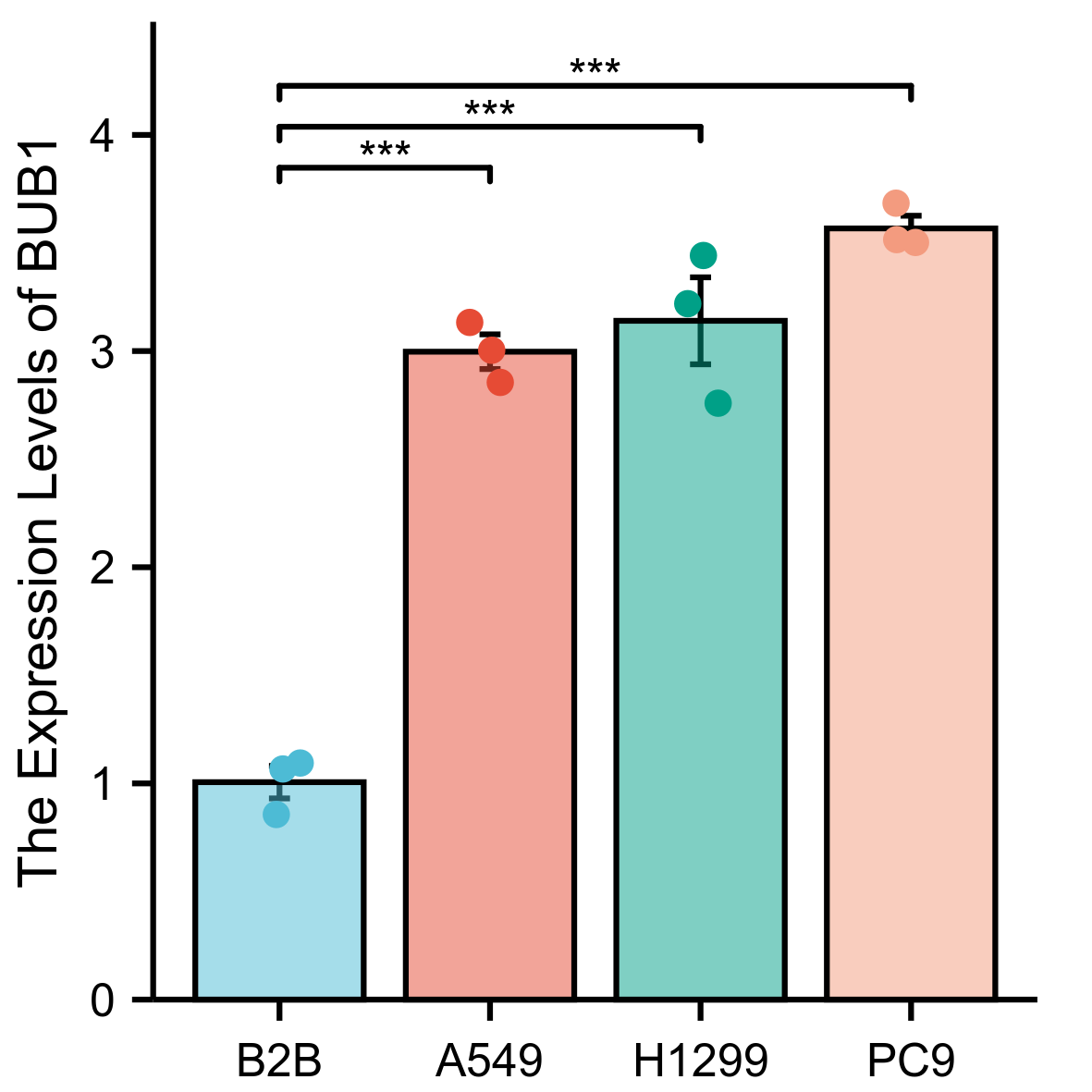

Supplement: Supplementary file 2 [file DataSheet2.zip › pcr/pcr图片(1)/BUB1.tiff]

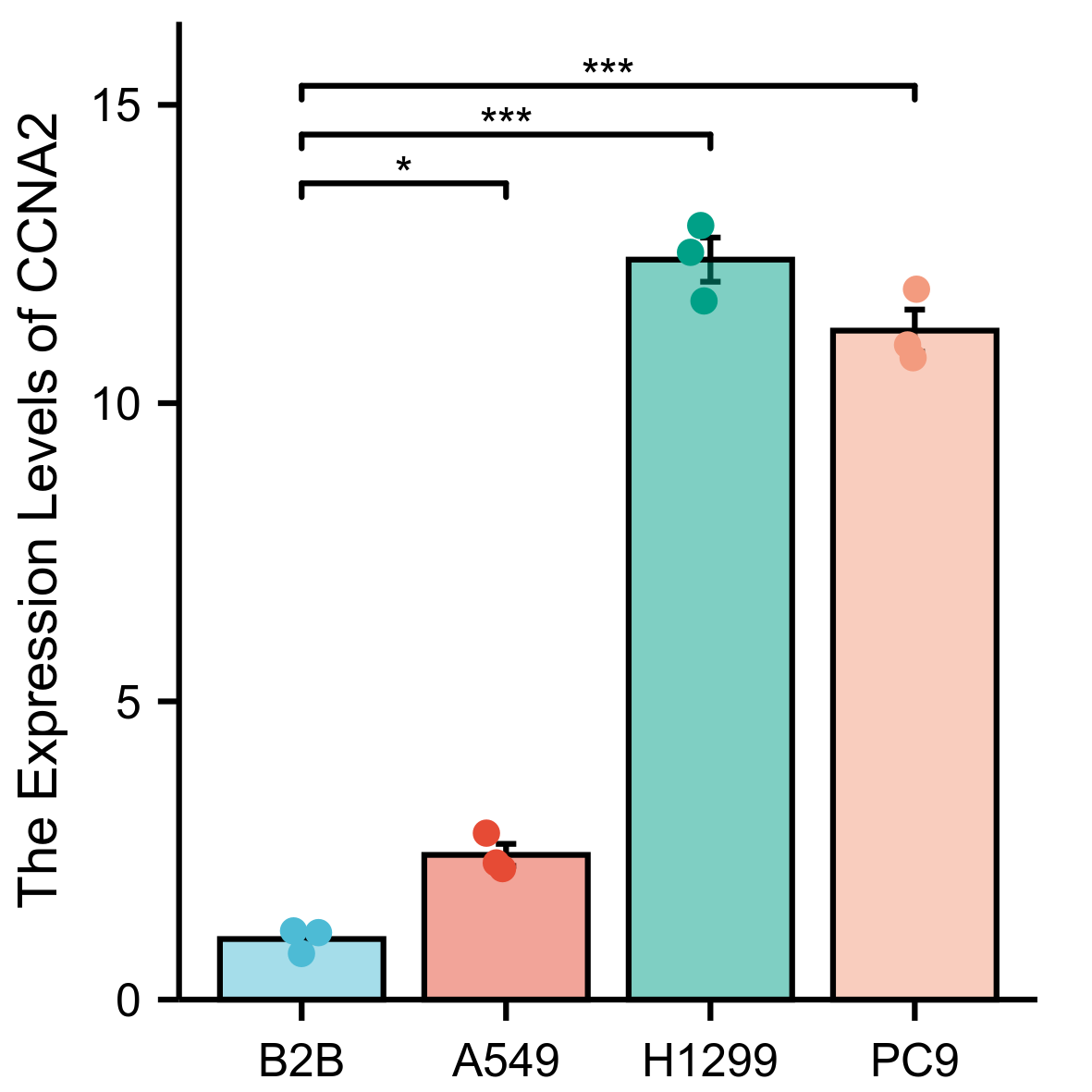

Supplement: Supplementary file 2 [file DataSheet2.zip › pcr/pcr图片(1)/CCNA2.tiff]

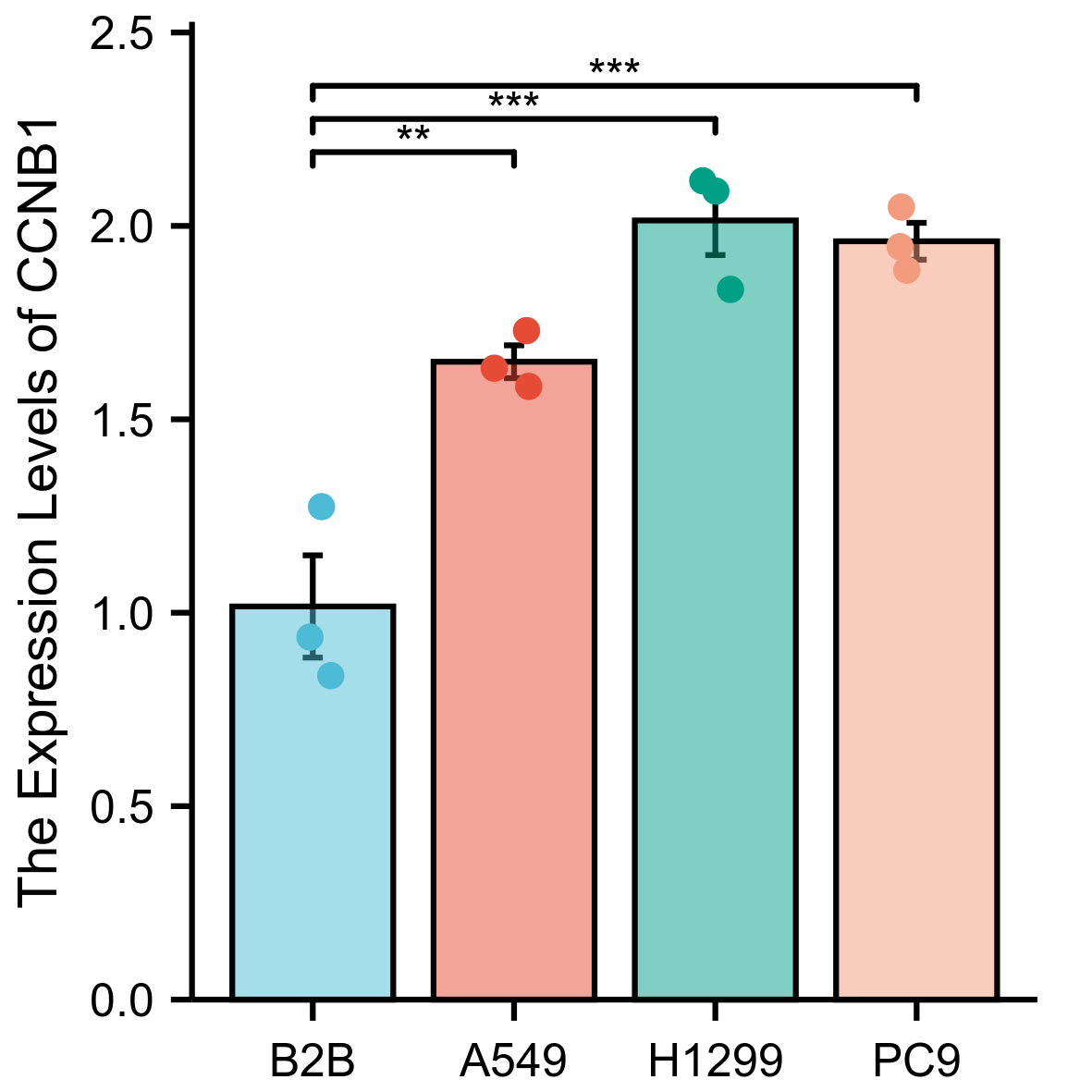

Supplement: Supplementary file 2 [file DataSheet2.zip › pcr/pcr图片(1)/CCNB1.tiff]

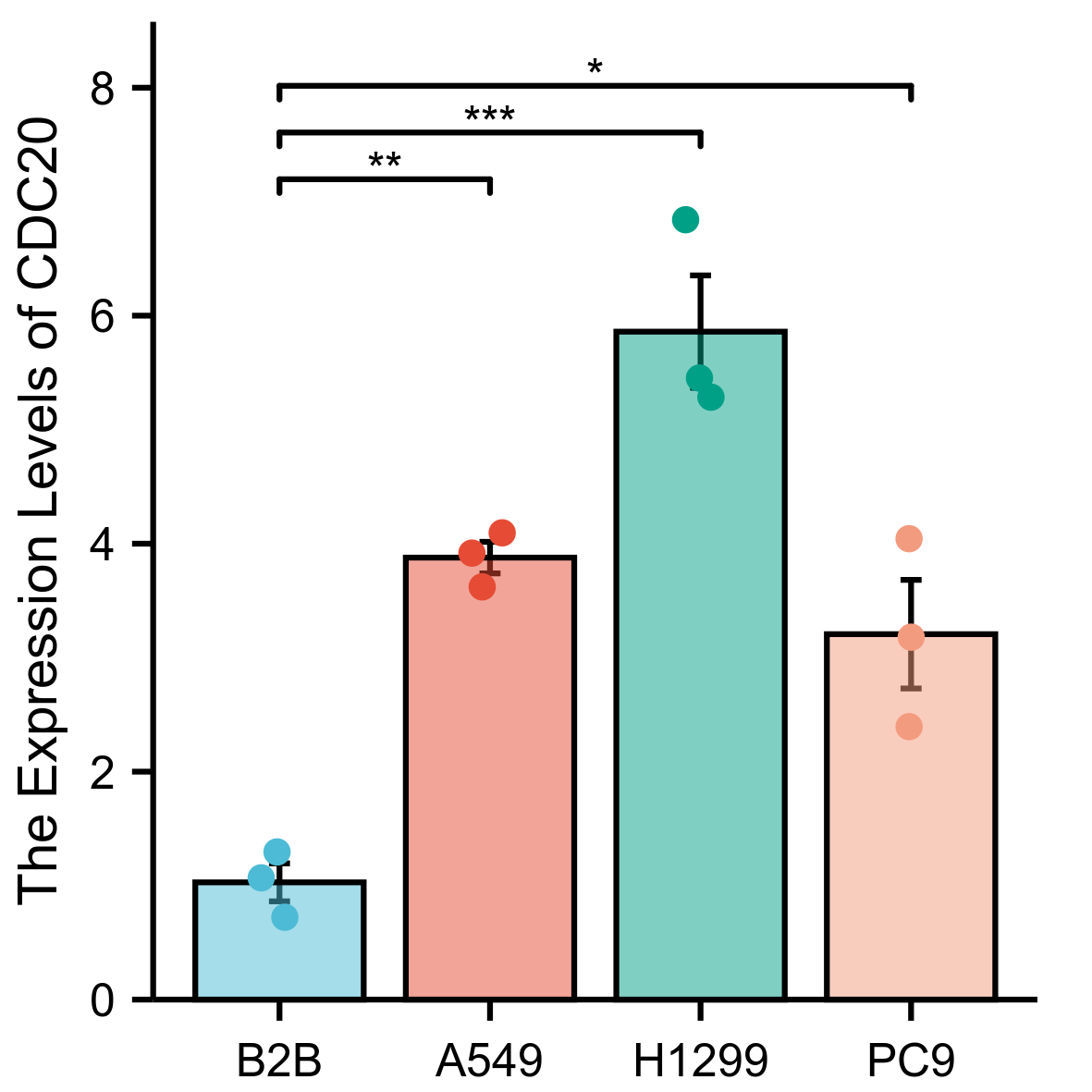

Supplement: Supplementary file 2 [file DataSheet2.zip › pcr/pcr图片(1)/CDC20.tiff]

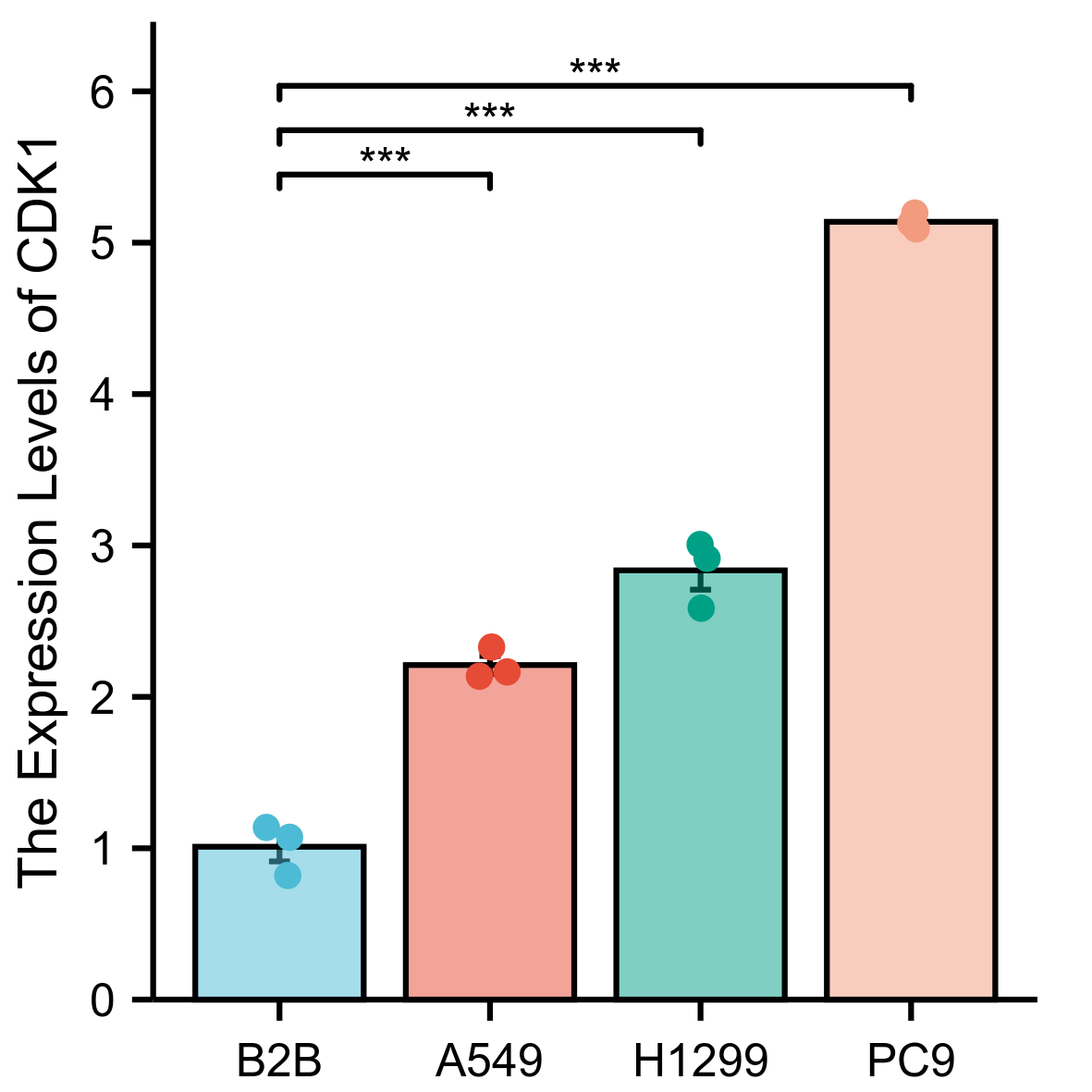

Supplement: Supplementary file 2 [file DataSheet2.zip › pcr/pcr图片(1)/CDK1.tiff]

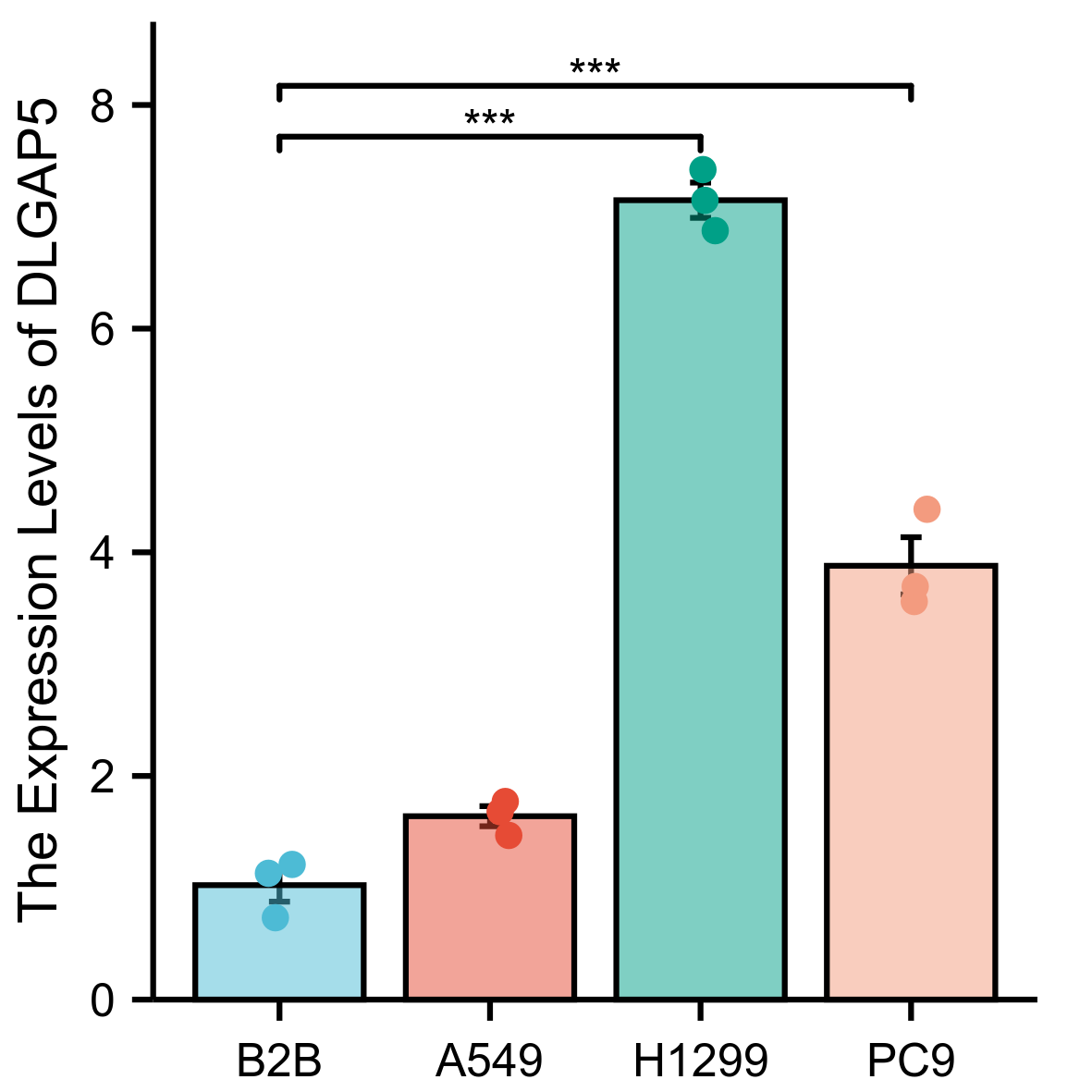

Supplement: Supplementary file 2 [file DataSheet2.zip › pcr/pcr图片(1)/DLGAP5.tiff]

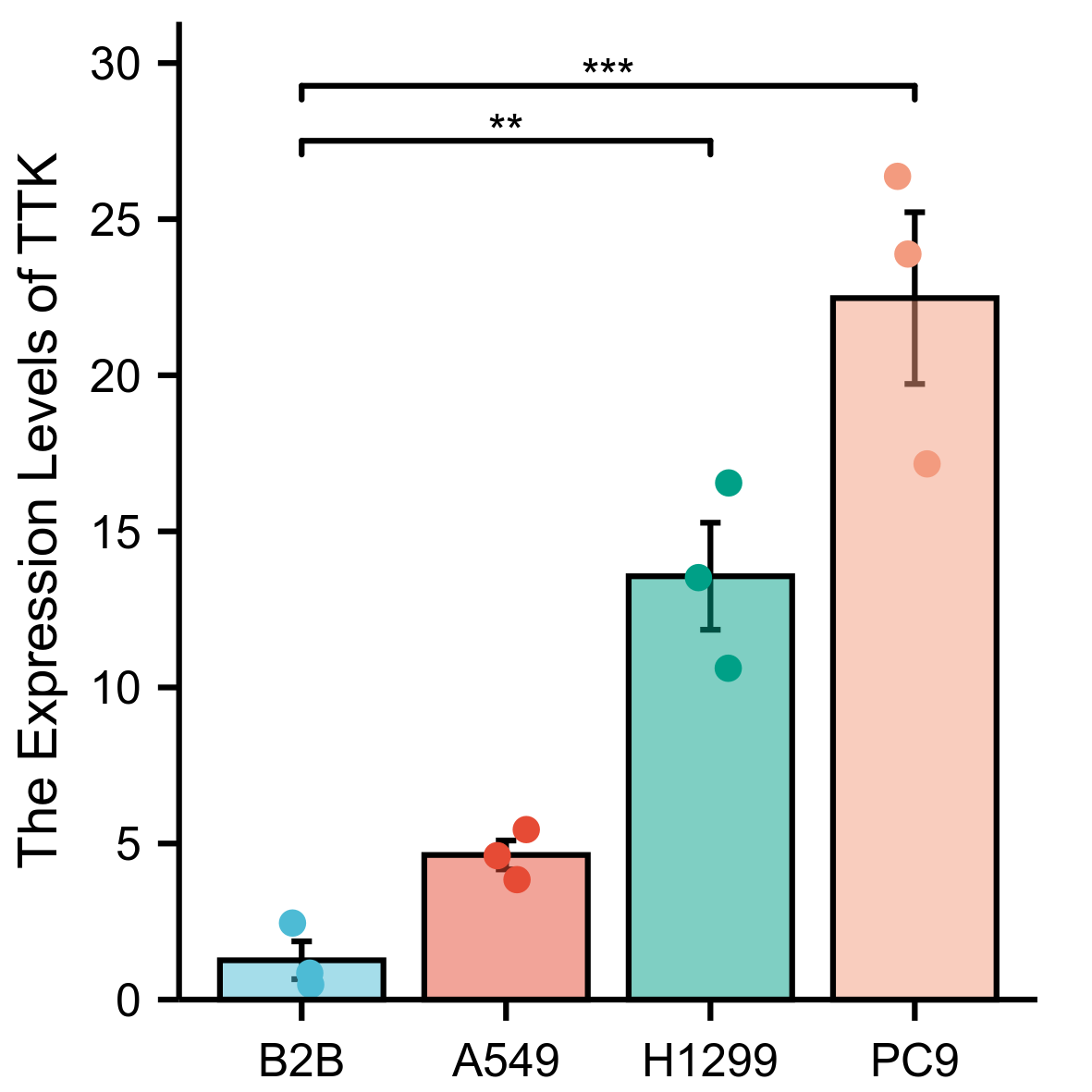

Supplement: Supplementary file 2 [file DataSheet2.zip › pcr/pcr图片(1)/TTK.tiff]

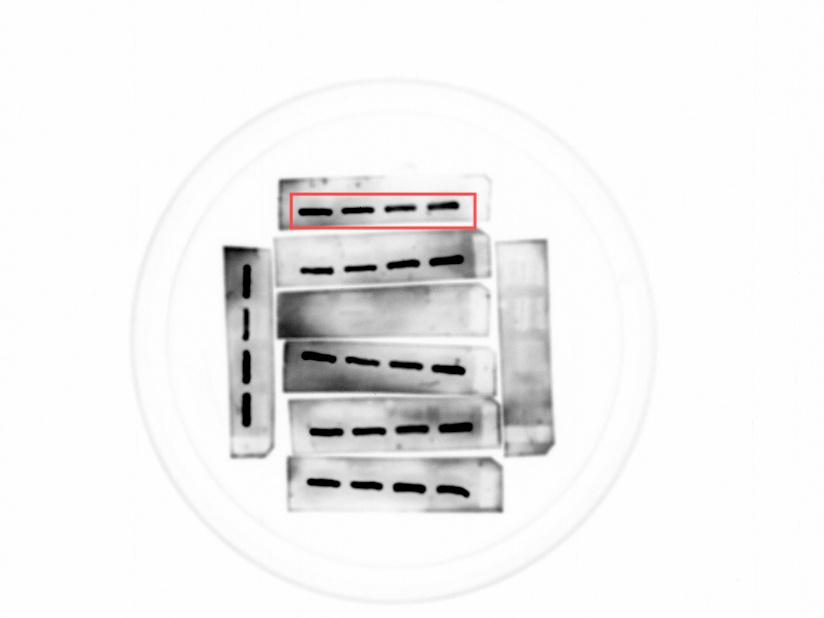

Supplement: Supplementary file 2 [file DataSheet2.zip › wb/Protein band/actin 1.jpg]

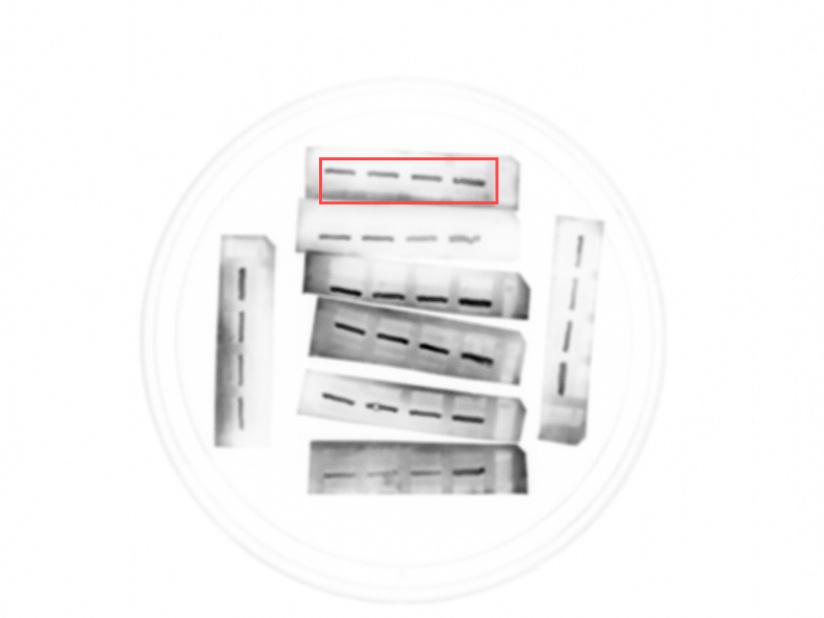

Supplement: Supplementary file 2 [file DataSheet2.zip › wb/Protein band/actin 2.jpg]

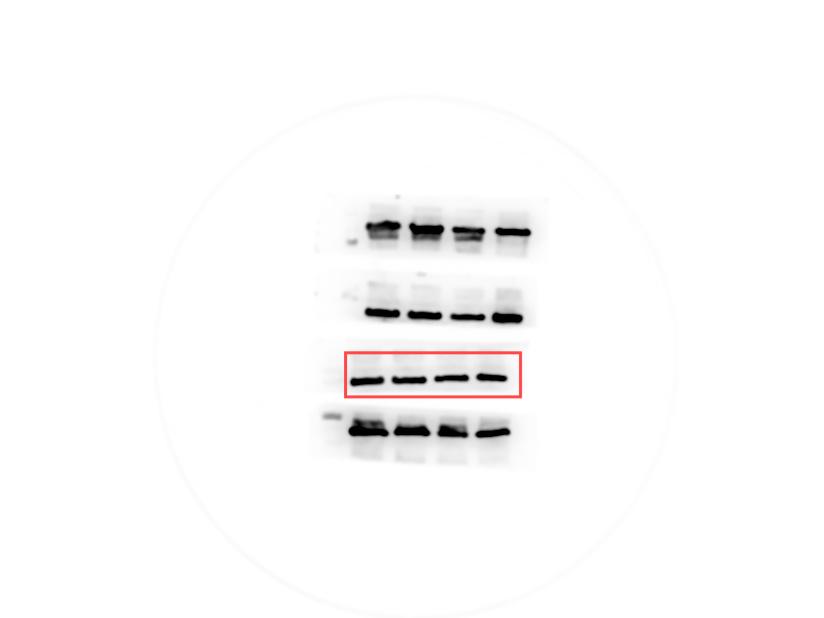

Supplement: Supplementary file 2 [file DataSheet2.zip › wb/Protein band/actin 3.jpg]

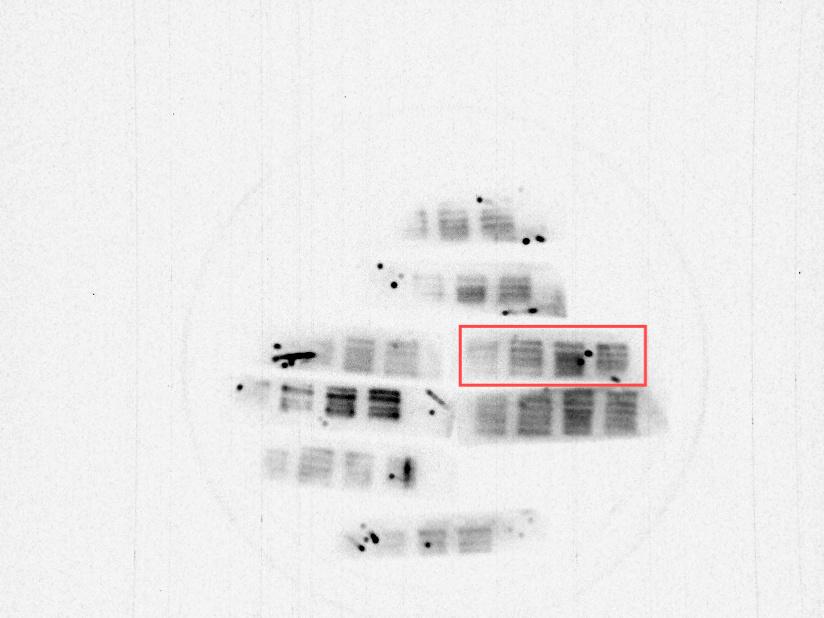

Supplement: Supplementary file 2 [file DataSheet2.zip › wb/Protein band/cdk1 1.jpg]

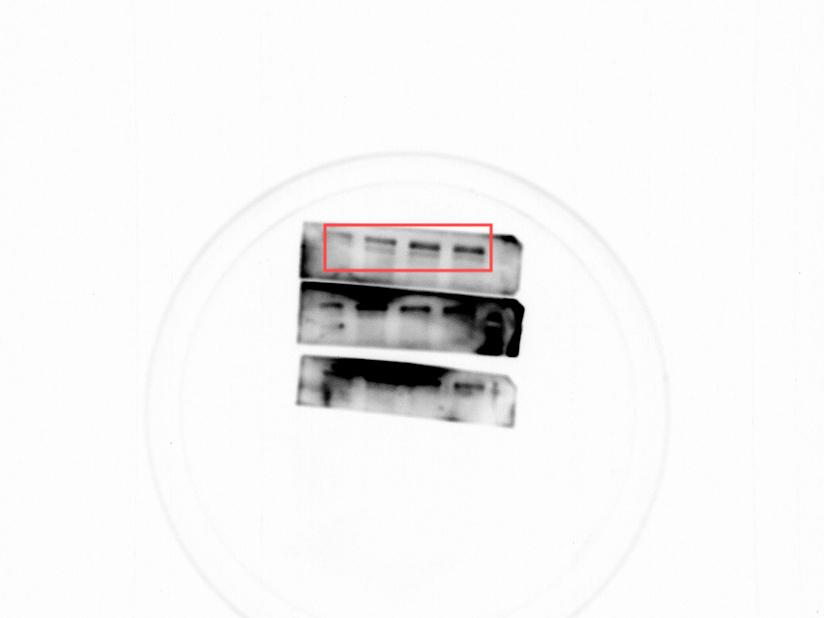

Supplement: Supplementary file 2 [file DataSheet2.zip › wb/Protein band/cdk1 2.jpg]

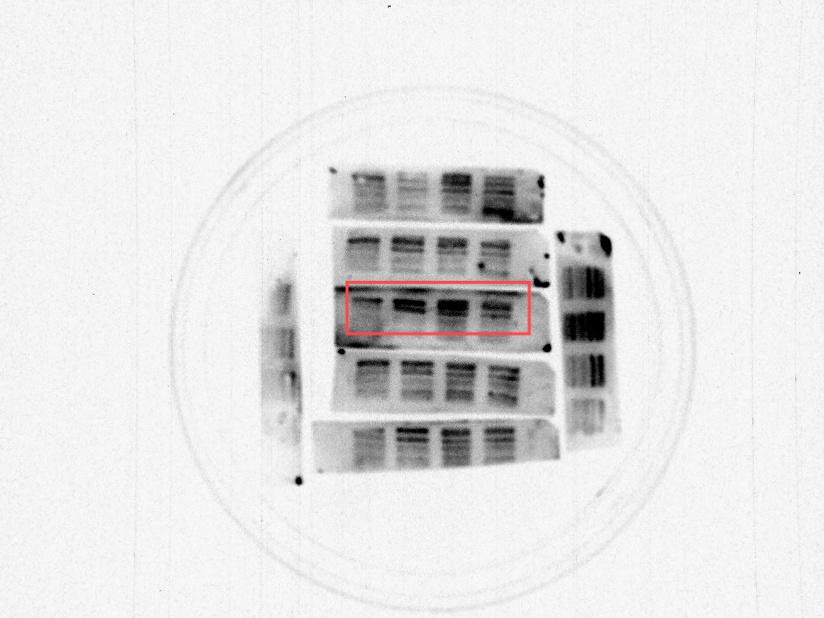

Supplement: Supplementary file 2 [file DataSheet2.zip › wb/Protein band/cdk1 3.jpg]
